# All‐Polyimide‐Mediated Liquid Metal Assembly on Aerogels for Breathable and Robust Electronic Skins

**DOI:** 10.1002/adma.73751

**Published:** 2026-06-17

**Authors:** Haijun Zhu, Jiancheng Dong, Hao Qiu, Je Hyeong Kim, Xingyu Liu, Shiyin Lin, Mengting Zheng, Chang Zhou, Yuduo Zhang, Jiayu Hou, Kangjia Geng, Yuchen Wang, Yidong Peng, Haoran Liu, Guanzheng Wu, Yunpeng Huang, Yongsheng Luo, Steve Park, Tianxi Liu

**Affiliations:** ^1^ Key Laboratory of Synthetic and Biological Colloids School of Chemical and Material Engineering Ministry of Education Jiangnan University Wuxi China; ^2^ Department of Materials Science and Engineering Korea Advanced Institute of Science and Technology (KAIST) Daejeon Republic of Korea; ^3^ College of Textiles and Clothing Yancheng Institute of Technology Yancheng Jiangsu China; ^4^ Kidney Transplantation Unit The First Affiliated Hospital of Zhengzhou University Zhengzhou China

**Keywords:** electronic skin, health management, liquid metal, molecular engineering, polyimide aerogel

## Abstract

Constructing breathable electronic skins (E‐skins) that maintain high electrical performance remains a formidable challenge due to the difficulty of establishing robust conductive networks on fragile porous substrates. To address this issue, a robust bio‐interface based on an ultralight polyimide aerogel that combines high vapor permeability (1700 g m^−^^2^ day^−^^1^) with intrinsic hydrophobicity (>123°) is developed. Leveraging the superior thermal stability (up to 460°C), a liquid metal particle ink encapsulated by polyamic acid shell is activated through mild thermal treatment at 140°C. The resulting imidization‐induced contraction generates compressive stress to rupture insulating oxide layers and achieve a high electrical conductivity of 8.17 × 10^5^ S m^−^^1^. This architecture employs an isotropic open‐cell framework that confines the ink to the surface while maintaining interconnected three‐dimensional (3D) pathways for lateral gas transport, thereby ensuring sustained breathability regardless of conductive layer coverage. Strong interfacial bonding through chemical coordination and mechanical interlocking enables negligible resistance variation over 200 000 deformation cycles and reliable underwater functionality. These synergistic advantages support diverse applications including conformal Joule heating, magnetic field sensing, and high‐fidelity electrophysiological monitoring during intense motion, establishing a versatile platform for next‐generation wearable healthcare.

## Graphical abstract

An all‐polyimide‐mediated assembly strategy resolves the fundamental conflict between physiological breathability and electromechanical robustness in wearable electronics. By integrating a polyamic acid‐encapsulated liquid metal ink onto an ultralight polyimide aerogel, imidization‐induced contraction enables low‐temperature conductive activation and robust interfacial anchoring. Because this surface‐confined architecture strictly preserves the underlying interconnected pore network for lateral gas transport, the platform delivers exceptional water vapor permeability and fatigue resistance to support durable high‐fidelity electrophysiological monitoring during continuous wear.

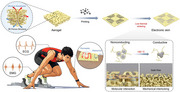

## 1 Introduction

Electronic skins (E‐skins) have garnered significant attention for their immense potential in continuously monitoring complex human kinematics and diverse physiological signals [1, 2], offering transformative solutions for healthcare management [3, 4], human‐machine interfaces [5], and soft robotics [6]. An ideal E‐skin must combine reliable signal transmission with comfort to allow seamless integration into daily life [7, 8]. However, most current substrates rely on dense and hermetical polymer films such as polydimethylsiloxane [9], polyurethane [10, 11], polyethylene terephthalate and polyimide (PI) [12, 13, 14]. Although these polymer films provide good flexibility, they suffer from inferior breathability (air permeability < 1 mm s^−^^1^, water vapor transmission rate < 50 g m^−^^2^ day^−^^1^), which is far below the insensible perspiration rate of human skin under sedentary indoor conditions (≈600 g m^−^^2^ day^−^^1^) [15]. Consequently, sweat accumulation at the device‐skin interface leads to irritation and signal degradation during prolonged use [16, 17].

To resolve this permeability issue, researchers have turned to porous substrates such as fibrous networks and polymer aerogels [18]. Among these options, robust PI aerogels offer a distinct advantage over mechanically weaker cellulose, gelatin alternatives, and fibrous mats [19, 20] due to their superior flexibility, thermal stability (>400°C) [21, 22], chemical resistance [23], and biocompatibility [24, 25]. These unique attributes establish PI aerogels as ideal platforms for durable and breathable electronics, yet transforming these porous insulators into functional devices remains a fundamental challenge. Specifically, functionalizing such architectures via printing method might compromise their breathability through pore blockage or results in poor interfacial adhesion due to the high porosity [26, 27].

To address these requirements, liquid metal particles (LMPs) offer a compelling strategy by enabling solution‐processable routes that leverage their high metallic conductivity and inherent softness [28, 29, 30]. This processability facilitates a seamless and monolithic integration within porous substrates, as the LMPs can effectively infiltrate the aerogel matrix to conformally coat its internal framework. This approach allows the conductive network to become one with the scaffold without compromising its intrinsic breathability. However, the insulating oxide (Ga_2_O_3_) shell surrounding these particles impedes electrical transport and often leads to weak interfacial bonding with flexible substrates [31, 32, 33]. Restoring conductivity typically necessitates aggressive mechanical or chemical activation steps that are incompatible with delicate aerogel structures [34]. Furthermore, achieving robust adhesion between heavy LMPs and lightweight aerogel substrates is difficult without compromising the bulk permeability of the three‐dimensional (3D) network. Therefore, the development of practical breathable electronics depends on finding a method to simultaneously achieve robust adhesion and mild electrical activation on porous PI substrates.

Herein, we report a robust strategy to fabricate breathable and highly compliant E‐skin via the synergistic integration of ultralight polyimide aerogels (PIA) and a molecularly engineered liquid metal particle ink. The isotropic PIA substrate is synthesized via sol‐gel polymerization of 3,3′,4,4′‐Biphenyltetracarboxylic dianhydride (BPDA) and 2,2′‐dimethyl‐[1,1′‐biphenyl]‐4,4‱‐diamine (DMBZ) monomers followed by freeze‐drying and thermal imidization. To functionalize this porous architecture, an electronic ink is formulated by encapsulating liquid metal microparticles within a polyamic acid (PAA) shell. Upon printing, the PAA shell undergoes in‐situ imidization under mild heating to trigger polymer chain contraction, which generates sufficient compressive stress to rupture the insulating oxide layers and activate the conductive network. This chemical conversion simultaneously facilitates strong intermolecular bonding with the aerogel framework while the isotropic open‐cell structure provides the robust skeletal support necessary to confine the conductive ink to the surface region. By maintaining a 3D interconnected network beneath the printed tracks, this architecture enables lateral gas transport that allows water vapor to effectively bypass the conductive pathways through unobstructed internal channels. Consequently, the resulting E‐skin preserves high breathability regardless of surface coverage and exhibits exceptional stability under rigorous mechanical deformation or underwater operation. We demonstrate the versatility of this platform through biocompatible devices ranging from tunable Hall sensors to reliable electrodes for long‐term electrophysiological monitoring. This work establishes a universal methodology for constructing durable and multifunctional E‐skins that successfully resolve the traditional conflict between breathability and structural reliability in wearable electronics.

## 2 Results and Discussion

### 2.1 Unique Features of Highly Flexible Polyimide Aerogel

**FIGURE 1:**
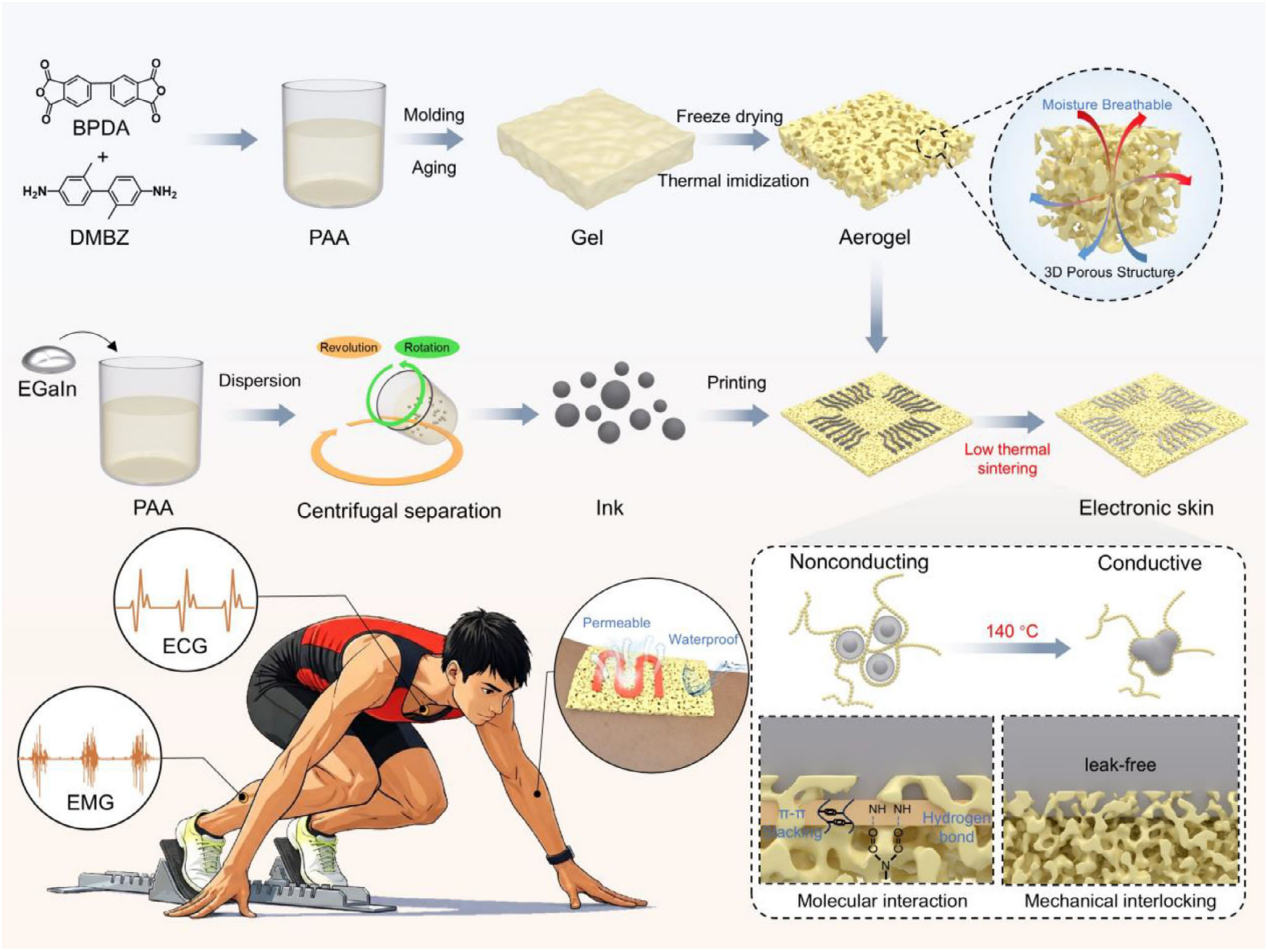
Fabrication process and application of PIA E‐skin.

**FIGURE 2:**
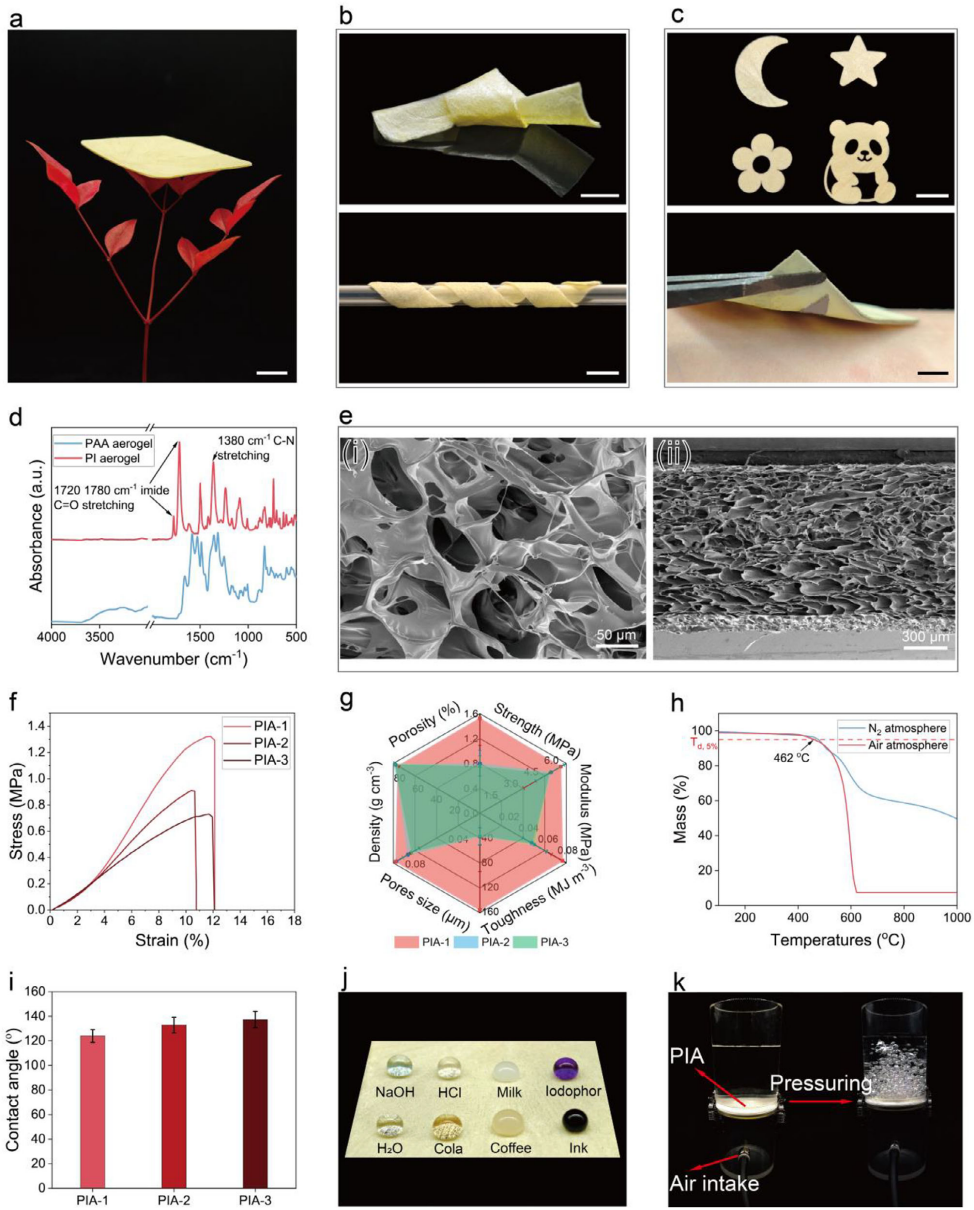
Digital images showing the (a) ultralight property, (b) high flexibility, and (c) customization and skin conformity of the PIA substrates (Scale bar: 1 cm). (d) FT‐IR spectra of PAA and PI aerogels. (e) SEM images of PIA prepared at freezing temperature of −20°C: (i) surface and (ii) cross‐sectional morphology. (f) Stress–strain curves and (g) comprehensive performance comparison of PIA‐1, PIA‐2 and PIA‐3 substrates. (h) TG curves of PIA under nitrogen and air atmospheres. (i) Water contact angle of PIA‐1, PIA‐2 and PIA‐3 substrates. (j) Hydrophobicity demonstration of PIA. (k) Air permeability demonstration of aerogels.

The schematic in Figure 1 illustrates the process of fabricating the permeable and flexible PIA E‐skin and its uses in electrophysiological monitoring, wearable Joule heating and motion control. Due to the molecular interactions and mechanical interlocking, highly conductive LM circuits can be firmly adhered to the porous aerogel substrate surface, facilitating long‐term robust conductivity. In addition, the open‐cell geometry enables water vapor to migrate laterally through the internal pores to bypass surface‐printed conductive tracks. This side‐permeability mechanism allows the system to maintain its high intrinsic breathability regardless of surface coverage as the underlying pores remain unobstructed, thereby maintaining thermal‐moisture balance between skin and environment [20]. The ultralight low density (≈0.09 g cm^−^^3^) of the PIA is vividly demonstrated by a 4 × 4 cm^2^ aerogel film resting on the tip of a leaf without bending the branch (Figure 2a; Figure S1a). Furthermore, the aerogel can be tied into a knot or rolled around a high‐curvature glass rod without mechanical failure (Figure 2b), which underlines the outstanding deformability of the porous substrate. The PIA films can also easily be laser‐cut to arbitrary geometries to tailor to specific applications, and the resulting PIA E‐skin fits smoothly to curved skin surface (Figure 2c), which highlights its capability to be used in skin‐interfaced electronics.

To convert PAA aerogels into PIA, the structure undergoes dehydration followed by cyclization (which is known as thermal imidization), as seen in Figure S2a. The FT‐IR spectra in Figure 2d reveal C═O stretching (1780 and 1720 cm^−1^) and C─N stretching (1367 cm^−1^), indicating imide linkages were formed successfully [35]. While this chemical conversion establishes the molecular backbone, the physical pore architecture is governed by the freezing kinetics employed during fabrication. At low temperatures, ice crystals serve as sacrificial templates, which maintain the porous network during sublimation [36, 37]. To investigate the effect of freezing temperatures on the porous structure, three distinct samples were prepared and designated as PIA‐1, PIA‐2, and PIA‐3, corresponding to temperatures of −20°C, −60°C, and −100°C, respectively. The PIA‐1 exhibited the largest pores with an average diameter of approximately 157 µm as shown in Figure 2e and Figure S1b. In contrast, decreasing the freezing temperature to −60°C for PIA‐2 and −100°C for PIA‐3 radically reduces the average pore size to about 38 µm and 37 µm, respectively (Figures S1b and S2). This trend can be associated with the classical nucleation theory where low temperatures cause rapid nucleation rate that can create a large number of small ice crystals before they grow bigger [38, 39]. These interfaces are a direct influence on the mechanical performance of the aerogel. The tensile strength of PIA‐1 has been reported to be 1.53 MPa, which is almost two times higher than that of PIA‐2 and PIA‐3, as seen in Figure 2f. The high mechanical strength of PIA‐1 is likely due to a thick solid skeletal structure that is able to withstand the mechanical load effectively. The negligible variation in pore size between PIA‐2 and PIA‐3 indicates that pore size reduction reaches a plateau at sufficiently low freezing temperatures, where rapid ice nucleation dominates and further decreases in temperature no longer produce smaller ice crystals. An effective comparison of porosity, tensile strength, Youngs modulus, toughness, pore size and density reveal that PIA‐1 is the appropriate substrate that balances between mechanical strength and structural integrity (Figure 2g; Figure S3). This mechanical resilience supplements the inherent thermal stability of the polyimide chain that is not degradable to temperatures up to 460°C in both nitrogen and air environments (Figure 2h; Figure S4 and Table S1).

Also, surface wettability experiments in Figure 2i indicate that PIAs are inherently hydrophobic in nature with water contact angles (WCA) surpassing 123°. The origin of this property has been traced to the methyl groups (‐CH_3_) of the aromatic rings of DMBZ that improve the level of molecular hydrophobicity [40]. In addition to water, the aerogel surface can be dewetting to a broad range of the popular liquids, such as cola, coffee, milk, ink, iodophor, and acidic/alkaline liquids (Figure 2j). Such a property prevents fouling or contamination during day‐to‐day operation, thus enhancing long‐term durability. Moreover, airflow experiments demonstrate that gas bubbles easily penetrate the PIA while submerged in water, proving its high air permeability as shown in Figure 2k and Movie S1. Combined, these findings make PIAs ultralight, flexible, thermally stable and permeable to moisture and best used in E‐skins.

### 2.2 Molecular Engineering of Liquid Metal Particle Inks for Low‐Temperature Activation

The exceptional mechanical flexibility and permeability of the PIA substrate provide an ideal foundation for electronic skin, yet the integration of conductive networks remains a fundamental challenge. Bulk LMs typically exhibit high surface tension and poor adhesion to porous surface [34, 41], which complicates precise deposition on aerogel structures. We addressed this limitation through a PAA‐assisted molecular engineering strategy that endows LM inks with tunable rheology and a unique contactless activation mechanism. This approach ensures the seamless integration of conductive pathways into polyimide aerogels for reliable device performance.

**FIGURE 3:**
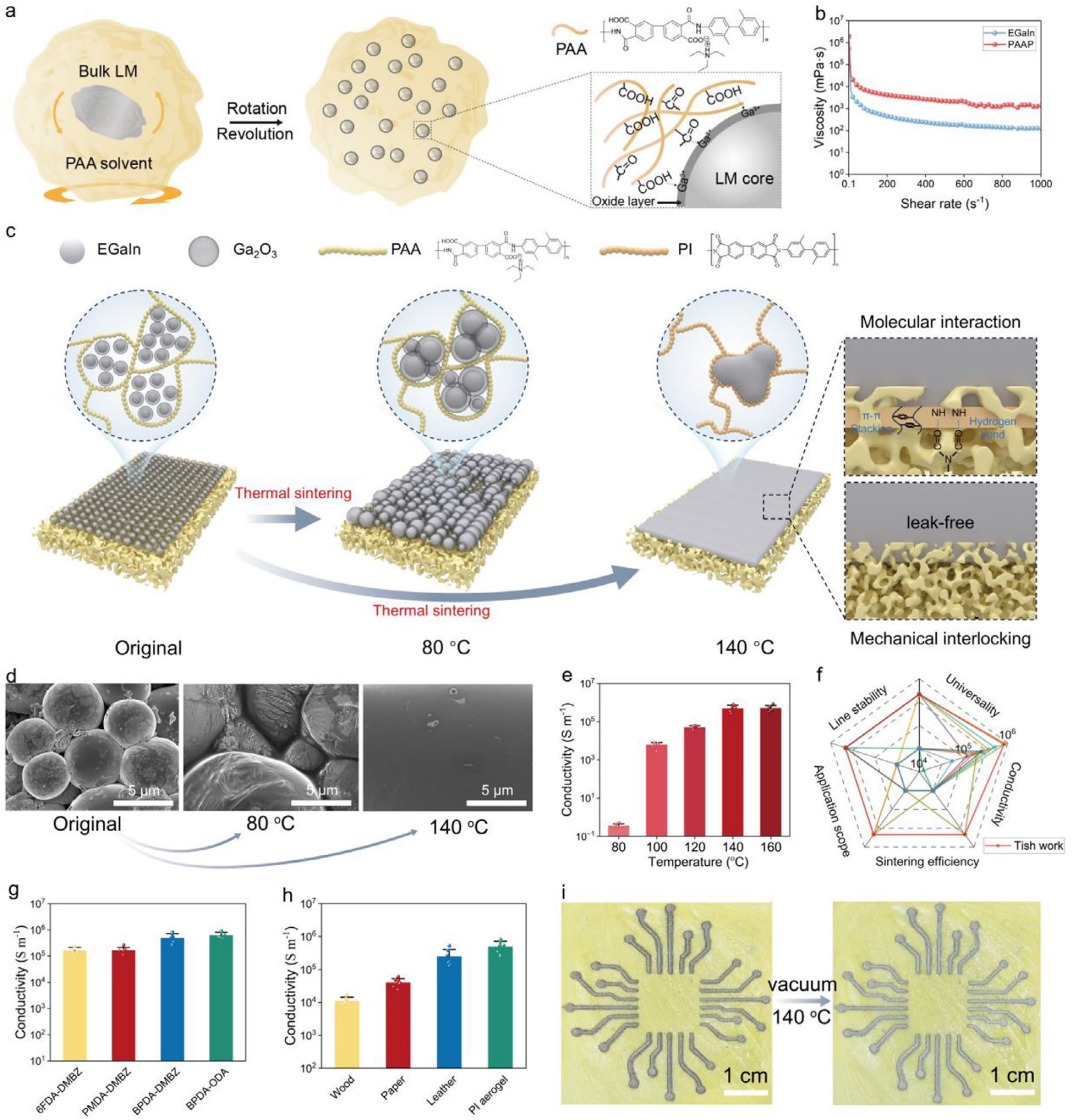
(a) Illustration of fabrication process of PAA‐encapsulated LM particle ink and molecular interactions between the PAA and LM particles. (b) Viscosity changes of the bulk liquid metal and PAAP. (c) Schematic diagram and (d) Corresponding SEM images of low temperature thermal sintering (80°C and 140°C) for PAAP. (e) Electrical conductivity of PAAP printed on PIA thermal sintered at different temperatures. (f) Comprehensive comparison of low‐temperature sintering with previously reported sintering strategies. (g) Electrical conductivity of PAAP prepared from another four representative PAA formulations after thermal sintering. (h) Electrical conductivity of thermally sintered PAAP printed on wood, paper, leather, and PIA substrates. (i) Patterns on polyimide aerogel before and after sintering.

Bulk liquid metal is dissipated into the PAA matrix by planetary mixing to produce PAA‐encapsulated microparticles (PAAP) with an average diameter of 1.7 µm (Figures S5 and S6). The carboxyl (‐COOH) and amide (‐CO‐NH‐) groups on the PAA chain coordinates with Ga^3^^+^ ions on the particle surface to form a robust organic‐inorganic interface that suppresses aggregation (Figure 3a) [42]. This formulation overcomes the inherent merging issues of pristine liquid metal by exhibiting optimized viscosity and printability (Figure 3b; Movie S2). When the ink is deposited over the PIA substrate, a robust interfacial bonding is formed by a bilateral stress on intermolecular hydrogen bonding and π‐π stacking, along with physical mechanical interlocking (Figure 3c). The ink partially infiltrates the surface pores to anchor the conductive network but does not permeate the entire aerogel thickness (Figure S7). This limited penetration is attributed to the rheological confinement of the viscous PAA shell and the physical barrier formed by the intricate aerogel pore walls (Figure S8). Crucially, this surface‐confined integration preserves the underlying isotropic open‐cell architecture which serves as a 3D lateral gas transport highway. Consequently, water vapor effectively bypasses the conductive tracks through the interconnected pore network to maintain high permeability.

Because of the high thermal stability given to the PIA substrate, a thermal activation strategy can be employed which would otherwise cause degradation of the traditional polymer with low thermal stability. Mild heating above 80°C triggers the imidization and subsequent volumetric contraction of the PAA shell (Figures S9 and S10 and Note S1) [43, 44]. This contraction is fundamentally driven by a sequential combination of physical and chemical processes along the heating profile. Initially, the evaporation of the residual organic solvent during the early stages of thermal treatment contributes to a preliminary macroscopic volume reduction [45, 46]. As the temperature increases, the thermal imidization of PAA into PI proceeds via dehydration cyclization where the elimination of water molecules intrinsically dictates the structural contraction of the polymer network [47, 48]. Furthermore, upon the formation of the PI backbone, strong intermolecular interactions inherent to polyimides such as charge transfer complex formation and preferred layer packing draw the rigid polymer chains closer to further promote matrix densification [46]. This mechanism is comprehensively supported by the newly added molecular dynamics simulations and experimental shrinkage analysis in Note S2 which quantitatively confirm that the PAA‐to‐PI conversion is accompanied by substantial polymer compaction and volume reduction. The resulting shrinkage generates intense compressive stress that fractures the insulating oxide layers and forces the liquid metal cores to coalesce into continuous conductive pathways (Figure 3c,d; Figure S11) [49]. The electrical conductivity increases by orders of magnitude between 0.37 S m^−1^ at 80°C and 6.32 × 10^3^ S m^−1^ at 100°C and 8.17 × 10^5^ S m^−1^ at 140°C with an effective sintering dosage (liquid‐metal particle loading) of 50 mg cm^−2^ (Figure 3e; Figure S12). Notably, pure LMPs inks remain insulating under identical conditions, highlighting the indispensable role of the PAA shell in stress‐driven activation (Figure S13). Unlike conventional approaches that require destructive mechanical sintering or high‐temperature annealing (>400°C) [50], this low‐temperature, stress‐driven activation strategy provides a mild, energy‐efficient, and contact‐free route for liquid‐metal sintering, while preserving structural integrity in delicate porous architectures and polymer‐integrated systems (Figure 3f and Tables S2; S3).

This activation mechanism is not limited to a particular set of monomers. Three additional exemplar PAA formulations, including BPDA–ODA, PMDA–DMBZ, and 6FDA–DMBZ also enabled successful formation of conductive networks under the same thermal conditions (Figure 3g; Figures S14 and S15). The ink also exhibits wide‐ranging application capability since it can be used on a wide range of substrates like wood, paper, and leather resulting in high conductivity (Figure 3h; Figure S16). The pattern on complex circuitry of using stencil printing on PIA‐based materials switched between black gray stage and metallic silver when activated to indicate the creation of the liquid metal phase (Figure 3i; Figure S17). Taken together, these findings permit sustainable solution of LM circuits on porous aerogels on a large scale, and thermal stability. The synergistic encapsulation provided by PAA, combined with low‐temperature activation, enables conformal, durable, and multifunctional PIA‐based E‐skins that can be seamlessly integrated into the structural and functional design of human skin.

### 2.3 Electromechanical Reliability and Multifunctional Wearable Applications

To further elucidate the electrical characteristics and structural reliability of the PAAP ink, we systematically evaluated its performance on both stretchable and flexible substrates. While the primary focus of this work is the integration of PAAP with highly flexible polyimide aerogels, additional measurements on elastomeric substrates were conducted to decouple the intrinsic electrical behavior of the ink from substrate effects and to highlight its strain‐insensitive conductivity, as detailed in Note S3.

**FIGURE 4:**
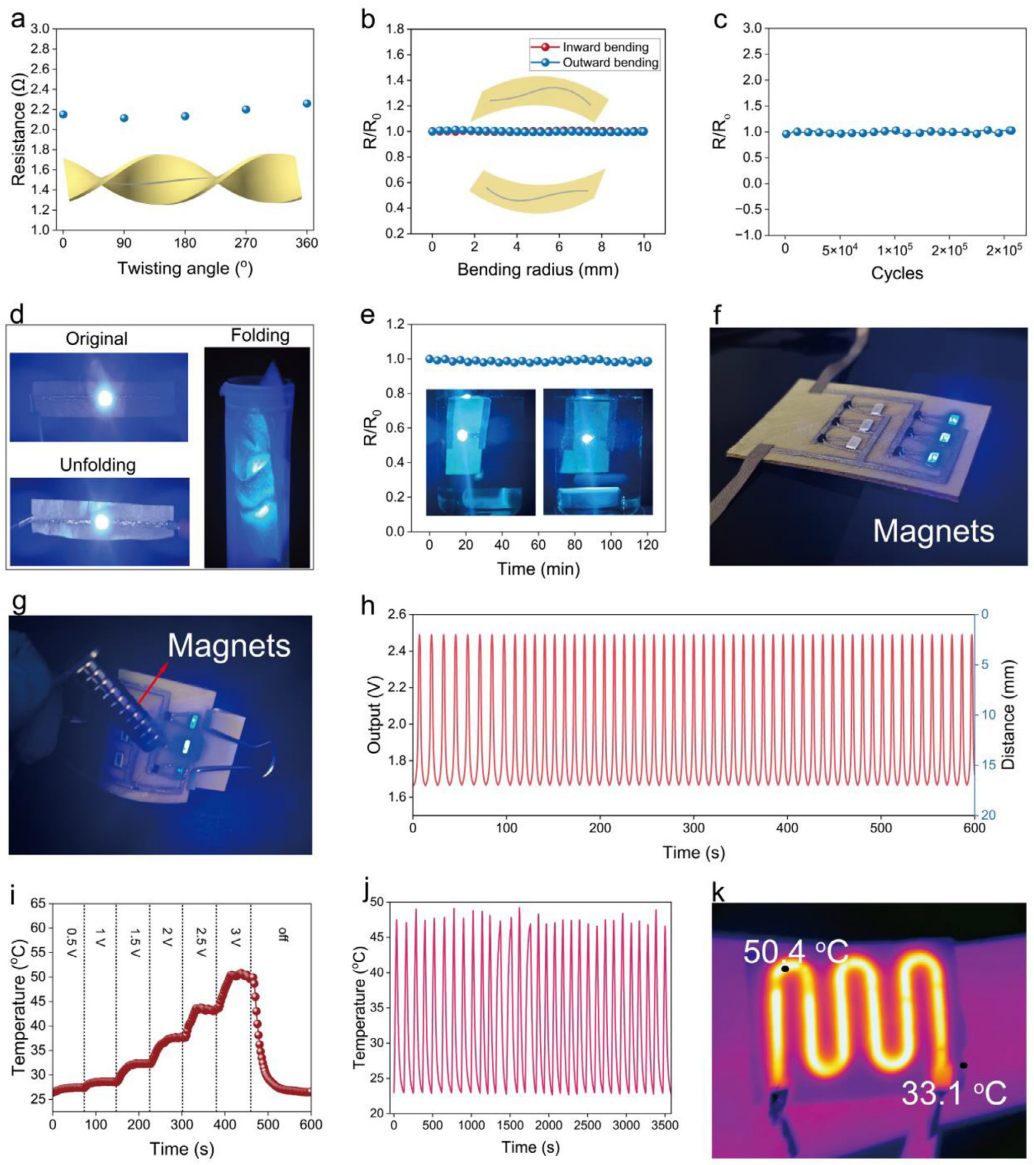
(a) Torsional resistance of the flexible CIPA conductor. (b) Resistance changes during inward and outward bending. (c) Relative resistance variation during 200 000 continuous inward bending cycles (bending radius: 2–10 mm; bending rate: 48 mm min^−^^1^). Demonstration of conducting stability of CIPA conductor against (d) complex mechanical deformations and (e) underwater disturbance (stirring speed: 300 rpm) when lighting a blue LED. Digital images of (f) Hall sensor device and the LED lighting up as the magnetic field changes and (g) the LED lighting stability after S‐shaped bending. (h) Output voltage versus magnetic field strength (distance: 2–20 mm; LED voltage threshold: 2 V). (i) Stepwise temperature rising curves at 0–3 V. (j) Durability of the Joule heating performance after 3600 s of heating‐cooling cycles under 3 V. (k) Practical application demonstration of the aerogel heater on the forearm.

Having established the intrinsic reliability of PAAP, it was then integrated onto highly flexible aerogel substrates to fabricate highly conductive polyimide aerogels (CIPA). The porous structure and high toughness of PIA enable stable electrical performance even under repeated mechanical deformation. When subjected to repeated twisting between 0° to 360°, the resistance of CIPA increased by merely 6.86% at the highest degrees of bending (Figure 4a). Moreover, inward and outward bending with radii between 2 to 10 mm to represent realistic motions in the joint have induced small resistance change with the largest variation being 2% (Figure 4b). The circuits did not undergo fatigue degradation even after cyclic bending (bending radius: 2–10 mm; bending rate: 48 mm min^−^^1^) of 200 000 times (Figure 4c). This outstanding cycling stability is closely related to the unusual mechanical resilience of the PIA substrate, whose flexible yet sufficiently robust open‐cell framework can effectively accommodate repeated bending while maintaining structural continuity and stable conductive pathways. This stability is not only manifested on the polyimide aerogel substrate, but also can be observed in other substrates (i.e., leather and paper), where the electrical stability of PAAP is still evident (Figure S18). Moreover, the CIPA was connected to a blue LED, which was folded and crumpled. The LED did not dim during such deformation with no electrical failure (Figure 4d). Moreover, due to its inherent hydrophobicity (WCA > 120°), the CIPA retained full functionality even after 120 min of water immersion, with no observable change in resistance or LED brightness (Figure 4e). Further immersion tests in artificial sweat solutions with pH values of 4, 6 and 8 prepared using phosphate buffered solutions and in water elevated to 50°C under continuous mechanical stirring for 120 min showed negligible resistance variation as detailed in Figure S19. This sustained electrical performance originates from the intrinsic chemical inertness and hydrophobicity of the polyimide matrix which prevents corrosive ion penetration and further demonstrates the exceptional environmental stability of the platform under chemically and thermally relevant conditions.

The combination of strain‐insensitive conduction on elastic substrates and fatigue‐resistant performance on PIA demonstrates the versatility of PAAP for diverse flexible and wearable electronic systems. Two representative prototypes, including a Hall‐effect sensor and a conformable Joule heater, were developed to highlight the broad applicability of this system.

A flexible magnetic‐field sensing circuit was fabricated by printing PAAP interconnects on PIA films and integrating six Hall‐effect sensors with six blue LEDs as visual indicators (Figure S20). The sensor array was designed to convert magnetic‐field‐induced voltage variations into light signals. Without an external magnetic field, the output voltage remained near half of the 3.5 V input, which is below the LED threshold (≈2 V), thus no emission occurred. When a magnetic field was applied, the Hall voltage increased beyond the threshold, triggering LED illumination (Figure 4f and Movie S3). Moreover, the circuit maintained stable functionality even under complex deformation. Indicatively, bending in the form of S‐shape, sensing and optical indication showed continuity, which proved mechanical‐electrical decoupling factor inside the CIPA network (Figure 4g). Further, the magnetic flux density was adjusted by varying the magnet sensor distance (20 to 2 mm), which led to a progressive increase in the intensity of LED brightness and a linear relationship between the intensity of magical field and the Hall voltage (Figure 4h; Movie S4). These findings indicate that CIPA platform allows microelectronic components (once rigid) to be easily integrated into flexible systems and retain their signal stability, which opens up the path of soft and reliable magnetic sensing of electronic skins.

The CIPA can also be used as an effective electrothermal converter of on‐skin Joule heater. As depicted in Figure S21, the characteristics of U‐I curves followed Ohm's law precisely (R^2^ = 0.992), while the correlation between voltage squared (U^2^) and temperature rise fitted well with Joule's law (Q = U^2^/R, R^2^ = 0.993), enabling precise thermal control simply by adjusting the input voltage [51]. Under the applied direct current (DC) voltages of between 0.5 to 3 V, the electrothermal performance was studied in the duration of 7 min where the temperature of the surfaces was closely observed with the help of an ultrathin patch thermocouple. It is evident that surface temperature rose with applied voltage in a linear fashion (Figure 4i; Figure S22a). To evaluate long‐term stability, CIPA was subjected to cyclic heating and cooling at 3 V DC (Figure 4j). No significant variation was observed in the temperature profiles during 60 min continuous cycles, demonstrating excellent performance and reliability of the CIPA heater. With the added advantage of the inherent nature of the PIA substrate in terms of inherent flexibility and low thermal mass, the CIPA heater was able to be flat to conform to human skin (Figure 4k; Figure S22b) providing efficient, homogenous, and comfortable heat transfer.

### 2.4 Biocompatibility and Breathability of the PIA E‐Skin

**FIGURE 5:**
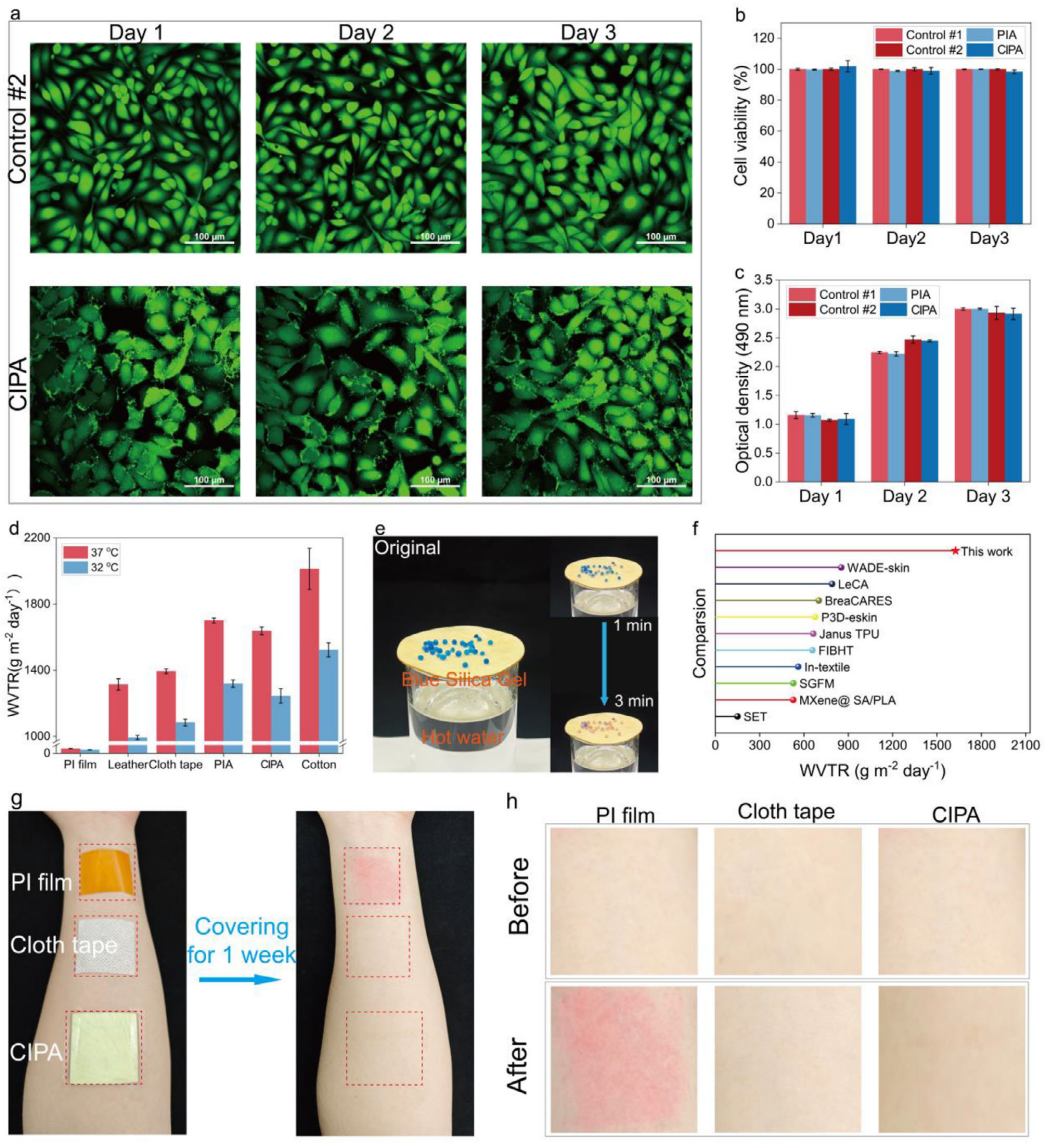
(a) Fluorescence images of cells cultured in a medium containing CIPA conductor and negative control group. (b) HUVEC viability and (c) Absorbance values at a wavelength of 490 nm in the MTT assay for different culture groups after 1–3 days of culture. (d) Comparison of WVTR values among PI film, leather, cloth tape, PIA, CIPA and Cotton at 32°C and 37°C. (e) Digital images demonstrating the rapid moisture permeability of the CIPA substrate via the visual transition of moisture sensitive silica gel from blue to pink. (f) Comparison of WVTR values with previously reported porous polymer substrates. (g,h) Digital images showing skin condition after a continuous 7‐day covering of PI film, cloth tape and CIPA on the forearm skin of a volunteer.

Biocompatibility is a prerequisite for wearable electronics and E‐skin applications [52]. In vitro cytocompatibility testing of HUVECs established that both PIA and CIPA are negligently cytotoxic. Live/dead fluorescence staining (Figure 5a) also indicated that cells were spread well with normal morphology and low numbers of dead cells as with the negative control. Cell viability measured quantitatively on day 1, day 2 and day 3 indicated that the cell viability among all our groups was not significantly different, with a cell viability not less than 94% in 3 days (Figure 5b). Regularly, MTT tests revealed that the absorbance increases proportionally with the viable cell counts over time and PIA group or CIPA group exhibited almost the same pattern as the control one (Figure 5c; Figure S23). The combination of all these results proves that PIA E‐skin has very high cytocompatibility meaning that it is safe to use it in long‐term epidermal application.

In addition to cytocompatibility, breathability is equally essential for maintaining skin health during continuous wearing. PIA E‐skin has remarkable moisture permeability which is shown by the water vapor transmission rate (WVTR) measurements in Figure 5d. A clear permeability gradient was observed across the samples, with PIA exhibiting the highest WVTR (1700 g m^−^^2^ day^−^^1^) owing to its open aerogel network, followed by CIPA (1637 g m^−^^2^ day^−^^1^), which retains high permeability despite complex LM pattern integration. In comparison, cloth tape and leather exhibited markedly lower WVTR, both below 1400 g m^−^^2^ day^−^^1^. Although these values are marginally lower than those of loose cotton textiles (2012 g m^−^^2^ day^−^^1^), they readily meet the perspiration requirements for human thermoregulation [15]. Conversely, PI film has many times less permeability (28 g m^−1^ day^−1^), underscoring the superior breathability of CIPA. Notably, CIPA maintains high WVTR values even at a lower temperature of 32°C (>1186 g m^−^^2^ day^−^^1^), indicating stable vapor transport under practical conditions. Even at high LM surface coverage ratios of 30%, 60%, and 90%, the WVTR remains at 1538, 1536, and 1524 g m^−^^2^ day^−^^1^ at 37°C, and 1247, 1225, and 1164 g m^−^^2^ day^−^^1^ at 32°C (Figure S24), respectively, confirming that the conductive layer causes only a limited reduction in breathability owing to the preserved three‐dimensionally interconnected pore network. Figure 5e further visualizes the excellent moisture permeability of the CIPA substrate through an indicator experiment using moisture sensitive silica gel. In this specific setup the rapid transport of water vapor through the conductive aerogel layer caused the dry blue silica gel to turn pink within 3 min. This compelling visual evidence directly corroborates the quantitative permeability data and confirms that the printed conductive network does not obstruct the internal gas diffusion pathways. Moreover, when compared to previously reported breathable E‐skins, CIPA has one of the highest WVTR values of all porous polymer‐based flexible substrates, which proves that it has a desirable breathability to comfortable E‐skin (Figure 5f; Table S4). A 7‐day continuous attaching on‐skin tests further validated its suitability for long‐term wearing (Figure 5g,h). Following the application of PI film on volunteer forearm, it produced redness and irritation, while CIPA and cloth tape left the skin unaffected. This is an indication that CIPA had high porosity and vapor permeability thus preventing sweat deposition and inflammation of the skin when in prolonged contact.

### 2.5 Long‐Term on‐Body Validation of Flexible PIA E‐Skin

**FIGURE 6:**
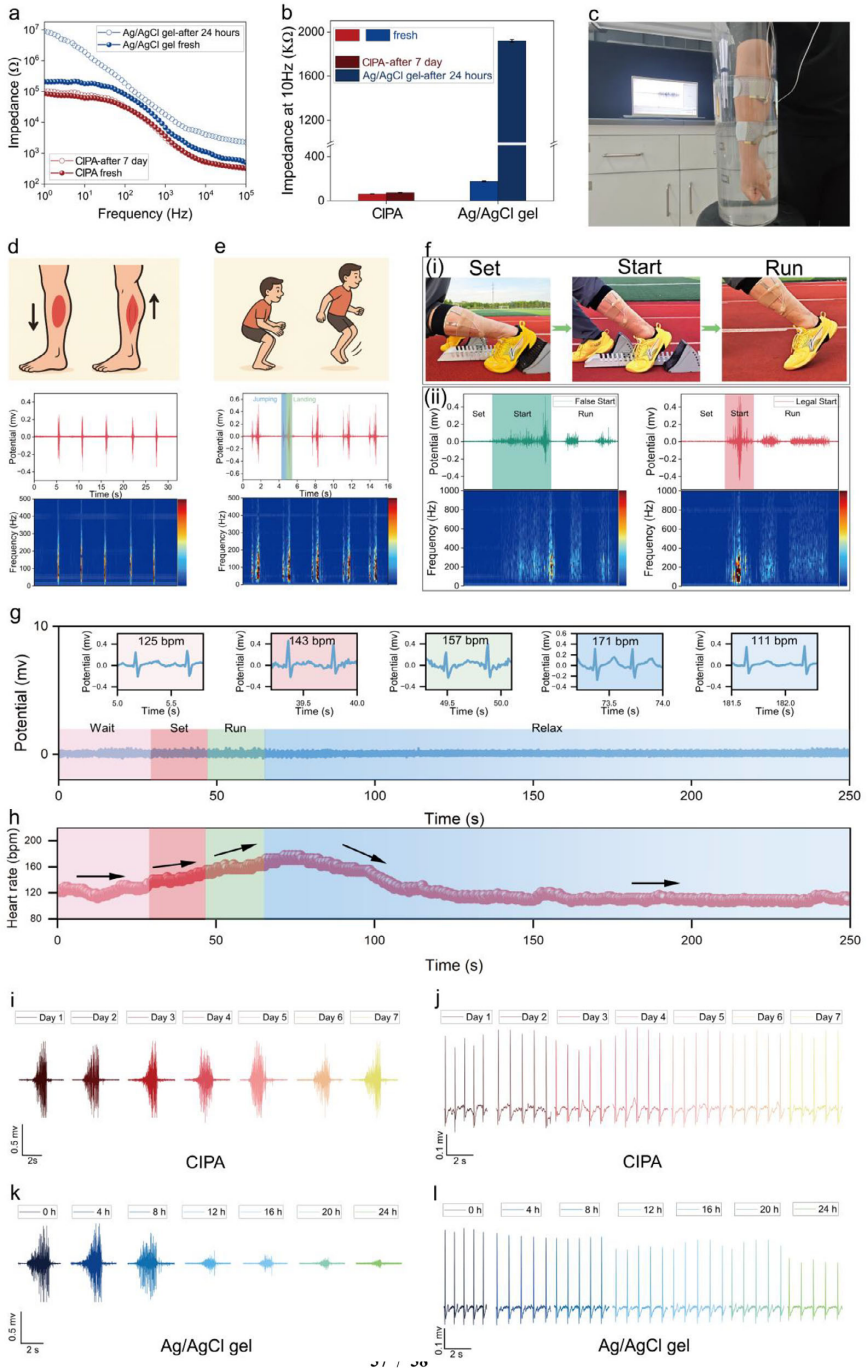
(a) Interfacial impedance comparison of the CIPA electrodes during a 7‐day continuous wearing versus the commercial Ag/AgCl electrode during a 24 h wearing. (b) Impedance comparison between the CIPA electrodes and commercial Ag/AgCl electrode at 10 Hz. (c) Digital image of underwater EMG recording with the aerogel electrodes. EMG signals recorded during (d) instantaneous tightening of the calf, (e) jumping and landing, and (f) set, start and run actions: (i) motion illustration and (ii) potential amplitude‐time and time‐frequency analysis. (g) Real‐time electrocardiogram (ECG) monitoring during a 100 m sprint. (h) ECG monitoring records. (i) Comparison of EMG signal recordings during fist‐clenching motions with the CIPA electrode under continuous wear for 7 days. (j) ECG signal recordings monitored by the CIPA electrode. (k) Comparison of EMG signal recordings during fist‐clenching motions with the Ag/AgCl electrode under continuous wear for 24 h. (l) ECG signal recordings monitored by the Ag/AgCl electrode.

The PIA E‐skin integrates high conductivity, flexibility, breathability, and excellent biocompatibility, thus making them ideal for continuous electrophysiological monitoring. As can be seen in Figure 6a, interfacial impedance of CIPA electrodes is significantly reduced as compared to Ag/AgCl versions in 1–10^5^ Hz spectrum, indicating more efficient charge transfer and enhanced signal sensitivity. Surprisingly, the electrodes of CIPA showed almost identical impedance after 7‐days of constant use, whereas commercial electrodes lost their quality after 24 h as a result of dehydration (Figure S25). At 10 Hz, the impedance of CIPA electrodes increased only 20% after 7 days, compared with a 1080% rise for Ag/AgCl electrodes within 24 h, further confirming its superior stability for long‐term biopotential recording (Figure 6b).

By integrating the CIPA electrodes with an elastic and breathable bandage, human electromyographic (EMG) signals can be reliably detected with excellent sensitivity (Figure S26 and Movie S5). At the same time, it can also operate normally in the aqueous environment thanks to its inherent hydrophobicity (Figure 6c; Figure S27). To systematically assess the responsiveness of the electrodes, we first examined lower‐leg muscle activation during a controlled explosive‐force test. When the calf muscle rapidly transitioned from a relaxed to a fully contracted state, the CIPA electrodes captured a burst of sharply localized EMG activity. Time–frequency analysis revealed a high‐intensity energy distribution concentrated within the physiological EMG band (0–500 Hz), with negligible low‐frequency motion artifacts, thereby confirming the high signal‐to‐noise ratio (SNR) and temporal resolution of the sensors (Figure 6d). To assess performance under more complex, dynamic conditions, EMG signals were recorded during repeated vertical jumping. Each jump involved alternating phases of ground contact, loading, and flight. Despite these pronounced dynamic perturbations, the EMG profiles consistently exhibited uniform bursts at the moment of push‐off. Time–frequency analysis further confirmed this stability, revealing distinct and repeatable spectral patterns that demonstrate the ability of the CIPA electrodes to retain high signal fidelity under highly nonstationary conditions and large accelerations (Figure 6e).

Following the validation of transient detection and dynamic stability, the system was deployed to record gastrocnemius muscle kinetics during competitive sprinting (Figure 6f). During sprint starts, muscles activate sequentially to generate propulsion, following a characteristic pattern of push‐off, thrust generation, and upward motion, which is reflected in distinct EMG bursts [53]. Per the World Athletics conventions of a false start, when the reaction to the starting gun occurs within 100 ms, it is counted as a false start [54, 55]. Nowadays, the starting blocks receive the starting gun, and the reaction to it indicates the false start. The reaction time is terminated once the initial block identifies that the force that is exerted by the athlete is beyond a set value. The size of this threshold has however the potential of causing delays in transmission of signal therefore impacting the correctness of the reaction time recorded [56]. EMG however is capable of analyzing muscle movements as early as 60 ms. A legal start produces high‐amplitude EMG spikes with sharp edges and a high spectral energy concentration as shown in Figure 6f, contrasting with a false start which has weaker signals scattered with a diffuse frequency distribution which implies premature and uncoordinated contraction [57]. These findings indicate that CIPA electrodes are capable of measuring transient muscle signals that are accurate and can be used to measure both performance and fault false‐start processing with greater accuracy in terms of time as compared to traditional force sensors.

In addition to the highly accurate detection of instantaneous triggers in the muscles, the system also supplements the continuous cardiovascular monitoring during high‐intensity physical exercise. To confirm this capability, we monitored real time electrocardiogram recording of a volunteer (through a 250 s window) to full 100 m sprint cycle (Figure 6g). The data captured contains four different stages such as waiting, set, running and relaxation. Figure 6h shows the extracted analysis of the heart rate and indicates a physiological pattern that is in perfect line in front of the changing physical requirements. This was because the heart rate ranged around 120 bpm in the waiting phase and was slightly increased during the set day because of anticipatory physiological arousal. This rose quickly after the start of the run in which the heart rate increased to about 160 bpm and sustained itself at that rate until the 16 s sprint duration ended. The nature of the exercise brought the heart rate to maximum heights around 170 bpm when the sprint was over. The relaxation phase was followed by subsequent monitoring that has revealed a slow decrease of the heart rate while the heart rate stabilized at around 110 bpm in the next 3 min of rest. This ability to read clear bioelectrical signals in the conditions of the violent body motions of sprinting is indicative of the superior contact stability of the CIPA electrodes.

Long‐term EMG and ECG monitoring further validated their reliability (Figure 6i–l). Over 24 h, commercial electrodes exhibited increasing baseline noise, amplitude attenuation, and periodic signal drift, while CIPA electrodes sustained clear, stable waveforms over seven consecutive days. Moreover, prolonged use of Ag/AgCl electrodes caused mild skin redness, whereas CIPA electrodes remained comfortable and irritation‐free (Figure S28). Quantitative analysis of signal quality revealed a distinct advantage in SNR and amplitude stability. For EMG recordings, commercial Ag/AgCl electrodes exhibited a rapid decline in performance: the SNR they had decreased to 10.2 dB in 24 h with a very large decrease in EMG amplitude (down to 0.05 mV). Conversely, its CIPA electrodes were capable of maintaining a high standard of recording fidelity during prolonged use, and the SNR did not reduce more than 34.2 dB on day 1 through 31.3 dB on day 7, although the amplitude of the EMG signal remained largely unaffected, at an average signal level of about 0.5 mV at each extreme end (Figures S29 and S30). The same thing was found in the case of ECG signals. Ag/AgCl electrodes indicated the decreasing SNR to 21.0 dB and also reduced signal amplitude as did the SNR to 0.15 mV. In contrast, the quality of signals obtained with CIPA electrodes was significantly higher, the SNR decreasing by a maximum of 30.1 dB to 26.7 dB but the amplitude itself was always stable (0.3 mV) and did not gradually decrease. All of these findings support the fact that CIPA electrodes have low impedance, high SNR, and very stable amplitude over long usage which allows the continuous and high‐quality monitoring of bioelectrical signals.

## 3 Conclusions

In summary, we have developed a breathable and mechanically robust electronic skin by integrating a polyimide aerogel with a molecularly engineered liquid metal ink. The substrate ensures wearable comfort through high moisture permeability and hydrophobicity. A critical innovation is the low‐temperature sintering strategy that activates conductivity at 140°C via imidization‐induced stress. This process ruptures oxide layers while anchoring the ink to the aerogel framework through robust chemical interaction and mechanical interlocking. Consequently, the composite conductor maintains stable electrical performance under extreme deformation and withstands over 200 000 bending cycles, even in underwater environments. These attributes directly enable versatile applications, including conformable Hall sensors, Joule heaters, and high‐fidelity electrophysiological monitoring during intense motion. This work presents a scalable paradigm for durable bioelectronics that effectively resolves the conflict between device reliability and long‐term user comfort.

## 4 Experimental Section

### 4.1 Materials

3,3′,4,4′‐Biphenyltetracarboxylic dianhydride (BPDA, 99.9%), 4,4′‐oxydianiline (ODA, 99.5%), 4,4′‐(hexafluoroisopropylidene) diphthalic anhydride (6FDA, 99.9%), 2,2’‐dimethyl‐[1,1′‐biphenyl]‐4,4′‐diamine (DMBZ, 99.5%), and pyromellitic dianhydride (PMDA, 99.5%) were purchased from Tianjin Zhontai Materials Technology Co., Ltd. N,N‐dimethylacetamide (DMAc, 99.0%) was obtained from Greagent, and triethylamine (TEA, 99.5%) was supplied by Adamas. The gallium–indium liquid metal alloy (Ga:In = 75:25) was purchased from Shenyang Jiabei Trading Co., Ltd.

### 4.2 Preparation of Polyimide Aerogel

Typically, 4.246 g (20 mmol) of DMBZ was added to a flask containing 40.52 g of DMAc and dissolved completely. Then, 5.884 g (20 mmol) of BPDA was added in portions. The mixture was stirred in an ice‐water bath for 6 h to yield a pale yellow poly (amic acid) solution. Subsequently, 2.5 mL of TEA was added dropwise, and the mixture was mechanically stirred for an additional 6 h to obtain a yellow poly (amic acid) salt solution (PAA). The product was precipitated in deionized water and freeze‐dried to obtain water‐soluble poly (amic acid) pulp. A 3 wt.% PAA solution was then prepared and frozen at −20°C, −60°C, and −100°C. The frozen samples were freeze‐dried for 48 h and then subjected to thermal imidization at 100°C, 200°C, and 300°C for 1 h to produce polyimide aerogels. The aerogels prepared from −20°C, −60°C, and −100°C freezing temperatures were designated as PIA‐1, PIA‐2, and PIA‐3, respectively.

### 4.3 Preparation of Liquid Metal Ink

The prepared PAA solution and liquid metal (gallium–indium alloy, 75:25) were mixed at a mass ratio of 4:1 using a planetary rotary mixer at 2000 rpm and then centrifuged. The supernatant was discarded to obtain PAA‐coated liquid metal ink. Before sintering, the liquid metal mass fraction in the ink was approximately 78 wt.%. After sintering, the liquid metal mass fraction increased to 98.9 wt.%, owing to solvent removal and thermal imidization of the PAA shell during the activation process. Under the corresponding printing conditions, the resulting conductive layer thickness was approximately 0.129 mm, which is consistent with the cross‐sectional morphology shown in Figure S8.

### 4.4 Characterizations

The morphology of the samples was examined using a field‐emission scanning electron microscope (Hitachi SU8600, Japan). The chemical structures were analyzed by Fourier transform infrared (FTIR) spectroscopy (Nicolet iS50, Thermo Fisher Scientific, Germany). The apparent density (ρ) of the aerogels was calculated from the geometric volume measured with a Vernier caliper and the mass determined using an analytical balance. Thermogravimetric analysis (TGA) was performed on a NETZSCH 209F1 instrument (NETZSCH, Germany) from 30 to 1000°C at a heating rate of 10°C min^−^^1^ under air and nitrogen atmospheres. The water contact angle was measured using a video‐based optical contact angle analyzer (Biolin Theta Flow). The mechanical properties of PIA were evaluated using a universal testing machine (MTS E42.503, MTS Systems China). The electrical performance of CIPA was characterized by collecting resistance data with a universal testing machine coupled to a benchtop digital multimeter (Tektronix DMM6500) at a compression speed of 48 mm min^−1^ conductivity was measured using a dual‐measurement digital four‐point probe system (Model ST2263, Suzhou Jing ge Electronics Co., Ltd.) The tested samples were CIPA strips with a width of 1 cm and a length of 5 cm. The thickness of the conductive liquid‐metal layer was controlled to 0.129 mm by the thickness of the printing stencil and was used for conductivity calculation. Infrared thermographic images and the corresponding temperature distributions were recorded using a Fluke TiS40 infrared camera (Fluke, USA). Conductivity was measured using a dual‐measurement digital four‐point probe system (Model ST2263, Suzhou Jingge Electronics Co., Ltd.) The tested samples were CIPA strips with a width of 1 cm and a length of 5 cm. The thickness of the conductive liquid‐metal layer was controlled to 0.129 mm by the thickness of the printing stencil and was used for conductivity calculation.

### 4.5 Electrophysiological Characterization

Biopotential signals (ECG and EMG) were collected by homemade recorders based on the Arduino platform. The signal‐to‐noise ratio (SNR) of the gel is calculated by the following formula, where N is the number of samples and V_signal(k)_ and V_noise(k)_ are the potential values of the signal and noise:

$$SNR\left(dB\right)=20\times log_{10}\frac{\sqrt{\sum_{k=1}^{N}V_{signal\left(k\right)}^{2}}}{\sqrt{\sum_{k=1}^{N}V_{noise\left(k\right)}^{2}}}$$

The experiments involving human participants were conducted exclusively for the technical evaluation of the wearable electronic skins on healthy volunteers. This research focuses entirely on materials development and device performance verification rather than medical clinical interventions. Consequently, clinical trial registration according to the International Committee of Medical Journal Editors guidelines is not applicable to this study. Appropriate institutional ethics approval was obtained prior to the experiments from the Ethics Committee of the First Affiliated Hospital of Zhengzhou University under the approval number 2026‐KY‐0811. Furthermore, informed written consent was successfully obtained from all human volunteers before their participation in the wearable device evaluations.

## Author Contributions

**H.Z**. and **J.D**. conceived the project and designed the overall architecture of the PIA–LM E‐skin system. **H.Z**., **H.Q**., **X.L**., **S.L**., **M.Z**., **Y. W**., and **C.Z**. fabricated the polyimide aerogels, prepared the PAAP inks, and carried out the device construction and characterization experiments. **J.H**. and **K.G**. performed the mechanical, electrical stability, and multifunctional device tests, including Hall sensing and Joule heating. **G. W**., **Y.L**., **Y.P**., and **H.L**. conducted the biocompatibility assays and moisture‐permeability measurements. **H.Z**. carried out the long‐term EMG/ECG on‐body evaluations and underwater monitoring tests. **J.D**., **T.L**., **Y.L**., and **Y.H**. supervised the project. **H.Z**. and **J.D**. analyzed the data and wrote the manuscript. All authors discussed the results and provided feedback on the manuscript.

## Conflicts of Interest

The authors declare no conflicts of interest.

## Supporting information




**Supporting File 1**: adma73751‐sup‐0001‐SuppMat.docx.

**Supporting File 2**: adma73751‐sup‐0002‐Movie S1.mp4.

**Supporting File 3**: adma73751‐sup‐0003‐Movie S2.mp4.

**Supporting File 4**: adma73751‐sup‐0004‐Movie S3.mp4.

**Supporting File 5**: adma73751‐sup‐0005‐Movie S4.mp4.

**Supporting File 6**: adma73751‐sup‐0006‐Movies S5.mp4.

## Data Availability

The data that support the findings of this study are available from the corresponding author upon reasonable request.
