# Supplementary material for: All‐Polyimide‐Mediated Liquid Metal Assembly on Aerogels for Breathable and Robust Electronic Skins
Source: Adv Mater. 2026 Jun 17;38(41):e73751. doi: 10.1002/adma.73751 (PMC13393982; doi:10.1002/adma.73751)
Supplement: Supplementary file 1 — Supporting File 1: adma73751‐sup‐0001‐SuppMat.docx. [file ADMA-38-e73751-s004.docx]

**Supporting Information**

**All-Polyimide-Mediated Liquid Metal Assembly on Aerogels for Breathable and Robust Electronic Skins**

Haijun Zhu, Jiancheng Dong*, Hao Qiu, Je Hyeong Kim, Xingyu Liu, Shiyin Lin, Mengting Zheng, Chang Zhou, Yuduo Zhang, Jiayu Hou, Kangjia Geng, Yuchen Wang, Yidong Peng, Haoran Liu, Guanzheng Wu, Yunpeng Huang, Yongsheng Luo, Steve Park*, and Tianxi Liu*

Haijun Zhu, Jiancheng Dong, Hao Qiu, Xingyu Lin, Shiyin Liu, Mengting Zheng, Chang Zhou, Yuduo Zhang, Jiayu Hou, Kangjia Geng, Yidong Peng, Haoran Liu, Yunpeng Huang, and Tianxi Liu

Key Laboratory of Synthetic and Biological Colloids, Ministry of Education, School of Chemical and Material Engineering, Jiangnan University, Wuxi 214122, China

Je Hyeong Kim, and Steve Park

Department of Materials Science and Engineering, Korea Advanced Institute of Science and Technology (KAIST), 291 Daehak-ro, Yuseong-gu, Daejeon, 34141, Republic of Korea

Yongsheng Luo

Kidney Transplantation Unit, The First Affiliated Hospital of Zhengzhou University, Zhengzhou 450052, China

Guanzheng Wu

College of Textiles and Clothing, Yancheng Institute of Technology, Yancheng, Jiangsu 224051, China

**Corresponding Authors**

Jiancheng Dong (email: [jcdong@jiangnan.edu.cn](mailto:jcdong@jiangnan.edu.cn))

Steve Park (email: [stevepark@kaist.ac.kr](mailto:stevepark@kaist.ac.kr))

Tianxi Liu (email: [txliu@jiangnan.edu.cn](mailto:txliu@jiangnan.edu.cn))

**The Supplementary Information file includes:**

Supplementary Note 1 to 3

Figures S1 to S30

Tables S1 to S4

Legends for Movies S1 to S5

**Other Supplementary Materials for this manuscript include the following:**

Movies S1 to S5

**Supplementary Note 1:**

To experimentally validate the hypothesis that volume shrinkage of the binder generates sufficient compressive stress to rupture the liquid metal oxide skin, we designed a comparative morphological study using scanning electron microscopy. A precursor dispersion was prepared by mixing polyamic acid solution and liquid metal at a mass ratio of 4:1 using a planetary mixer to ensure uniform encapsulation. This mixture was then subjected to two distinct thermal treatments under vacuum to isolate the effects of imidization-induced shrinkage.

In the first control group, the sample was dried at 80 °C. At this relatively low temperature, the solvent evaporation was the primary physical change, while the imidization reaction of the polyamic acid remained minimal. Consequently, the scanning electron microscopy images revealed that the liquid metal microdroplets remained intact and were conformally coated by the polymer matrix. The surface morphology appeared smooth with gentle protrusions, indicating that the polyamic acid shell successfully encapsulated the liquid metal cores without exerting significant mechanical compression (Figure NS 1a).

In stark contrast, the second group treated at 140 °C exhibited a fundamentally different microstructure (Figure NS 1b). This elevated temperature is known to trigger the thermal imidization of PAA into PI, a process accompanied by significant volumetric contraction. The resulting microstructure showed that the liquid metal was no longer smoothly encapsulated. Instead, numerous spherical liquid metal particles were observed emerging from the polymer matrix. This phenomenon suggests that the substantial shrinkage of the polymer shell generated intense compressive stress that squeezed the liquid metal droplets. This internal pressure forced the liquid conductive material to breach its encapsulating shell and the insulating oxide layer, leading to the extrusion and subsequent coalescence of the liquid metal. These morphological differences between the 80 °C and 140 °C samples provide direct visual evidence that thermal imidization induces the necessary compressive forces to activate the sintering process and establish conductive pathways.


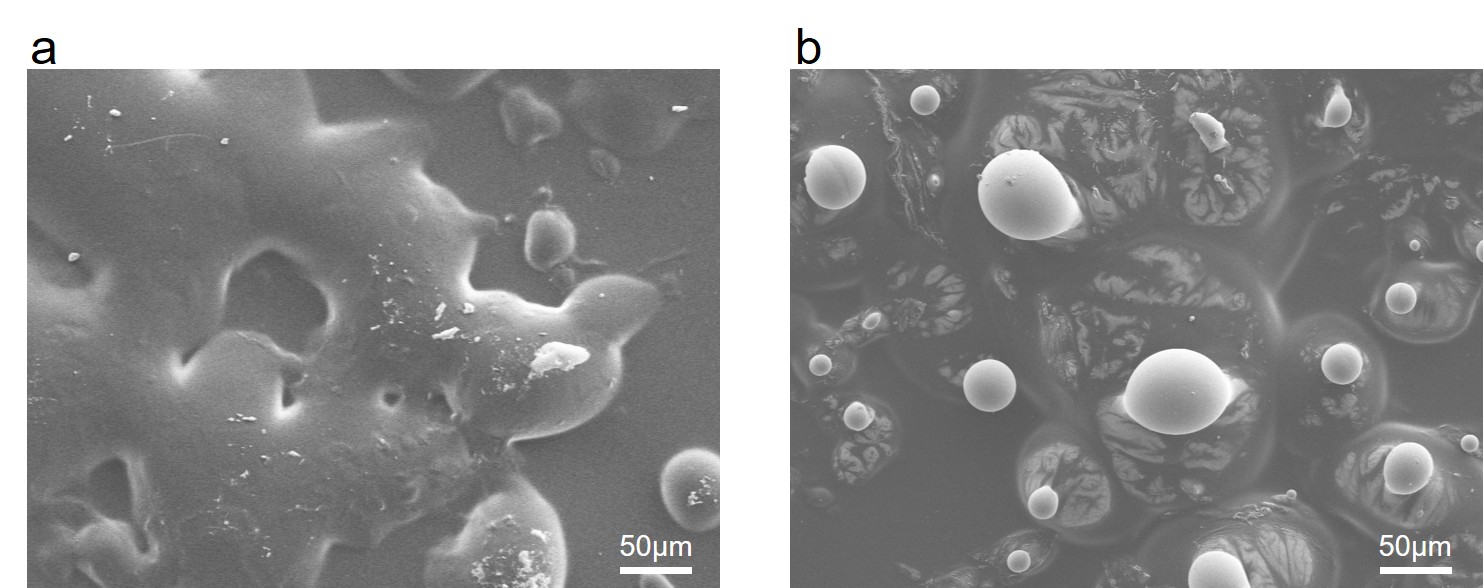


Figure NS1. (a) Corresponding SEM images of PAA and EGaIn dispersion at 80 °C. (b) Corresponding SEM images of PAA and EGaIn dispersion at 140 °C.

**Supplementary Note 2:**

**Coarse-grained molecular dynamics simulations**

The coarse-grained (CG) molecular dynamics (MD) model of polymer system was constructed. In the present CG MD model, a bead represents a cluster of atoms, involving plenty of repeat units. The PAA and PI molecules are modelled by a linear beads-spring chain consisting of *L*_P_ beads linked by *L*_P_-1 bonds, where that *L*_P_ is fixed at 24 without changing in the present work. The mass and diameter of each bead is set to be *m* and *σ*.

In the molecular dynamics (MD) simulations, the interaction potential includes the nonbonding potential *U_ij_* and bonding potential *U*_bond_. The nonbonding potential *U_ij_* is given by the modified Lennard-Jones 12:6 (LJ126) potential acting between any pair of *i*th and *j*th beads

$U_{ij}=\left\{ \begin{matrix} 4\varepsilon_{ij}\left[ \left( \frac{\sigma}{r_{ij}} \right)^{12}-\left( \frac{\sigma}{r_{ij}} \right)^{6}-\left( \frac{\sigma}{{r_{ij}}^{c}} \right)^{12}+\left( \frac{\sigma}{{r_{ij}}^{c}} \right)^{6} \right], r_{ij}\leq{r_{ij}}^{c} \\ 0, r_{ij}>{r_{ij}}^{c} \end{matrix} \right.$ (1)

where the *ε_ij_* is the interaction parameter between beads *i* and *j*. The *r_ij_*^c^ is the cutoff distance for *r_ij_* at which the potential is truncated and shifted to yield zero energy and force. In the modified LJ126 potential, the cutoff distance (*r_ij_*^c^) determines the attractive (*r_ij_*^c^ > 2^1/6^*σ*) or repulsive (*r_ij_*^c^ ≤ 2^1/6^*σ*) interaction between *i* and *j* beads, where the *σ* represents the distance unit in the MD simulations. To mimic the corresponding experimental system, the strength and cutoff distance for the interactions between beads *i* and *j* is fixed at 1.0*ε* and 2.5*σ* (attractive), respectively, where the *ε* represents the unit of energy in the MD simulations.

The bonding potential *U*_bond_ is given by the modified finite extensible nonlinear elastic (FENE) potential

$U_{\mathrm{bond}}=-0.5k_{b}{R_{0}}^{2}ln\left[ 1-\left( \frac{r}{R_{0}} \right)^{2} \right]$ (2)

where *k*_b_ = 20*ε*/*σ*^2^ and *R*_0_ = 1.5*σ* is the elastic coefficient and the maximum extensible bond length, respectively. We used a cosine harmonic function (angle potential) to further constrain the linearly rigid chain structure of polyimide, written as

$U_{\mathrm{angle}}\left( \theta\right)=\frac{1}{2}k_{a}\left( cos\theta-cos\theta_{0} \right)^{2}$ (3)

where *k*_a_ = 0 (for the flexible PAA) and 20*ε* (for the rigid PI) is the angle spring constant and *θ*_0_ = 180° is the equilibrium angle.

In the present MD simulation, the total number of CG MD beads is 48000, containing 2000 polymer (PAA or PI) molecules (each PAA or PI molecule chain includes *L*_P_ = 24 beads). All the MD simulations were carried out by the large scale atomic/molecular massively parallel simulator (LAMMPS), developed by Sandia National Laboratories.^[1]^ In the MD simulations, to generate the initial configurations, we constructed a large system with low volume fraction in a cubic box, which was compressed to the volume fraction of 0.45. Based on the initial configurations, the MD simulations were performed in the isothermal-isobaric (NPT) ensemble by using the Nose-Hoover barostat and thermostat. During the MD simulations, the periodic boundary conditions were imposed with a time step Δ*t* = 0.001*τ* (*τ* denotes the unit time).

**
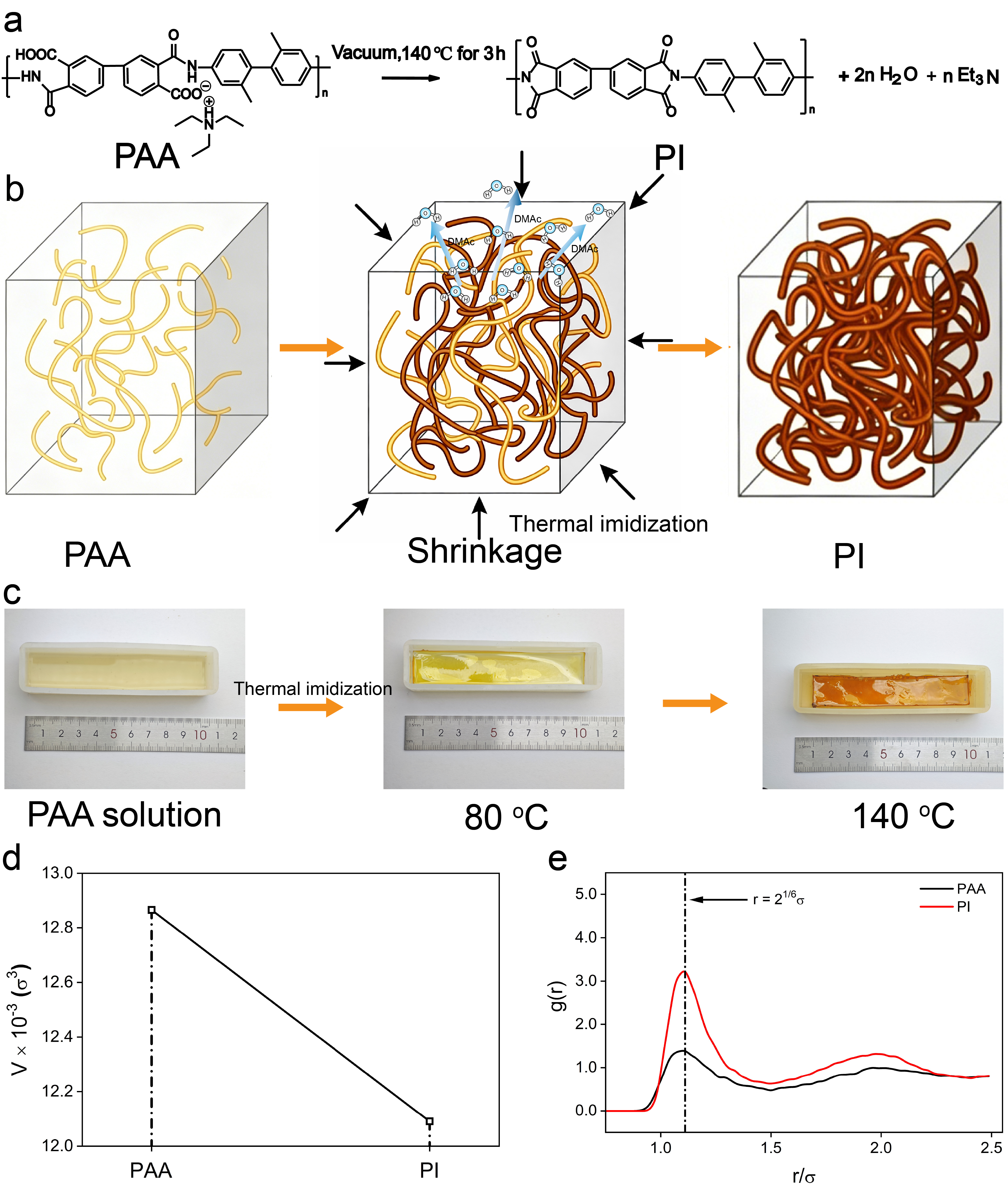
**

**Figure NS2.** (a) Chemical structure transformation from PAA (repeating units: n mol) to PI. (b) Schematic representation of the thermal imidization process where the solvent evaporation, water molecules elimination, and intermolecular interactions fundamentally drive the volumetric shrinkage of the polymer network. (c) Digital photographs capturing the initial PAA solution and the subsequent macroscopic states following thermal treatment at 80 °C and 140 °C. Coarse-grained molecular dynamics simulation of imidization from PAA to PI: (d) decrease in system volume and (e) radial distribution function.

**Supplementary Note 3:**

Liquid metals inherently possess infinite fluidity and deformability, which are favorable for maintaining conductive pathways under mechanical strain. To examine this property, PAAP was first printed on a highly stretchable VHB tape (Figure NS 3a). The resistance of the printed films was recorded in situ under uniaxial strain. In contrast to conventional metal or composite conductors that follow Pouillet’s law,^[2]^ the PAAP–VHB circuits maintained nearly constant conductivity even under extreme elongation. The normalized resistance (R/R₀) remained as low as 2.20 at 600% strain (Figure NS 3b), demonstrating the strain-insensitive nature of the PAAP network. Under cyclic stretching (100% strain for 10,000 s), the resistance fluctuation was less than 0.16 Ω (Figure NS 3c), confirming the robustness and dynamic stability of the encapsulated liquid metal network. Comparison with reported stretchable conductors further reveals that PAAP exhibits one of the smallest resistance variations at large strains （Figure NS 3d and Table S3), emphasizing its superior intrinsic electrical resilience.





**Figure NS3.** (a) Digital images of polyamic acid/liquid metal ink printed on VHB tape: lighting up at rest, lighting up under 200% stretching, and lighting up under 200% stretching with 270° twisting. (b) Resistance change rate of VHB tape and liquid metal under 600% stretching. (c) Conductivity stability test of VHB/liquid metal (cycled for 10,000 s under 100% strain). (d) Comparison of maximum stretching-resistance change rate.

**Supplementary Figures**


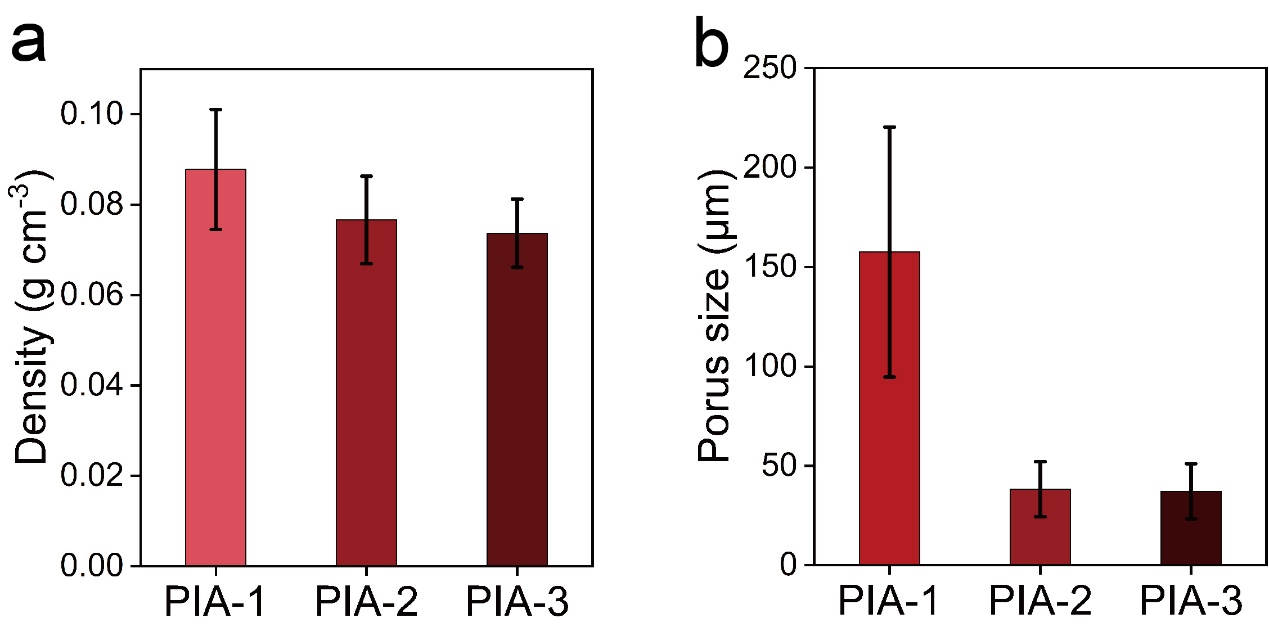


**Figure S1.** (a) Bulk density and (b) pore size comparison of polyimide aerogels.


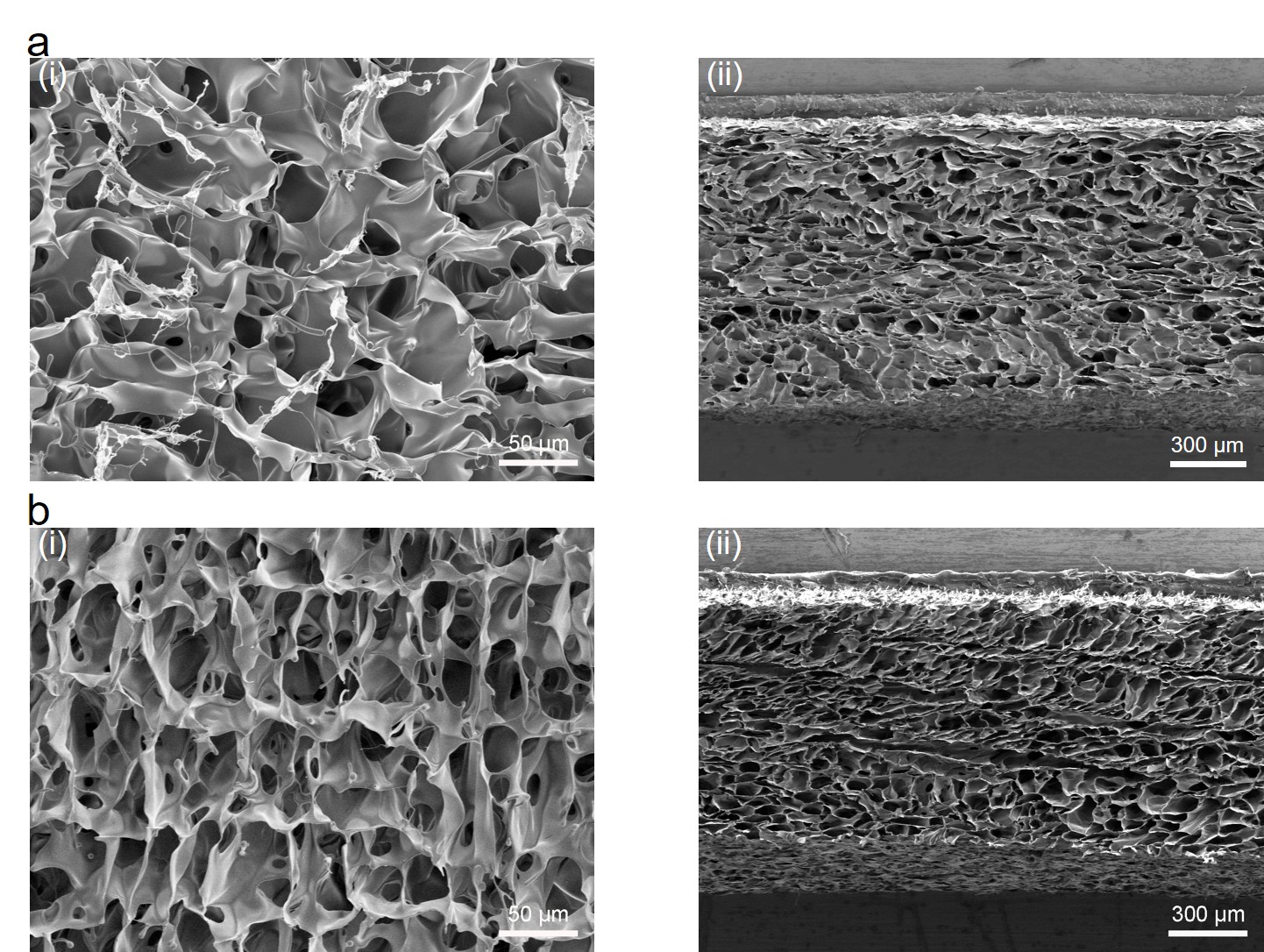


**Figure S2.** SEM images of PIA prepared at freezing temperature of (a) -60 °C, and (b) -100 °C: (i) surface and (ii) cross-sectional morphology.


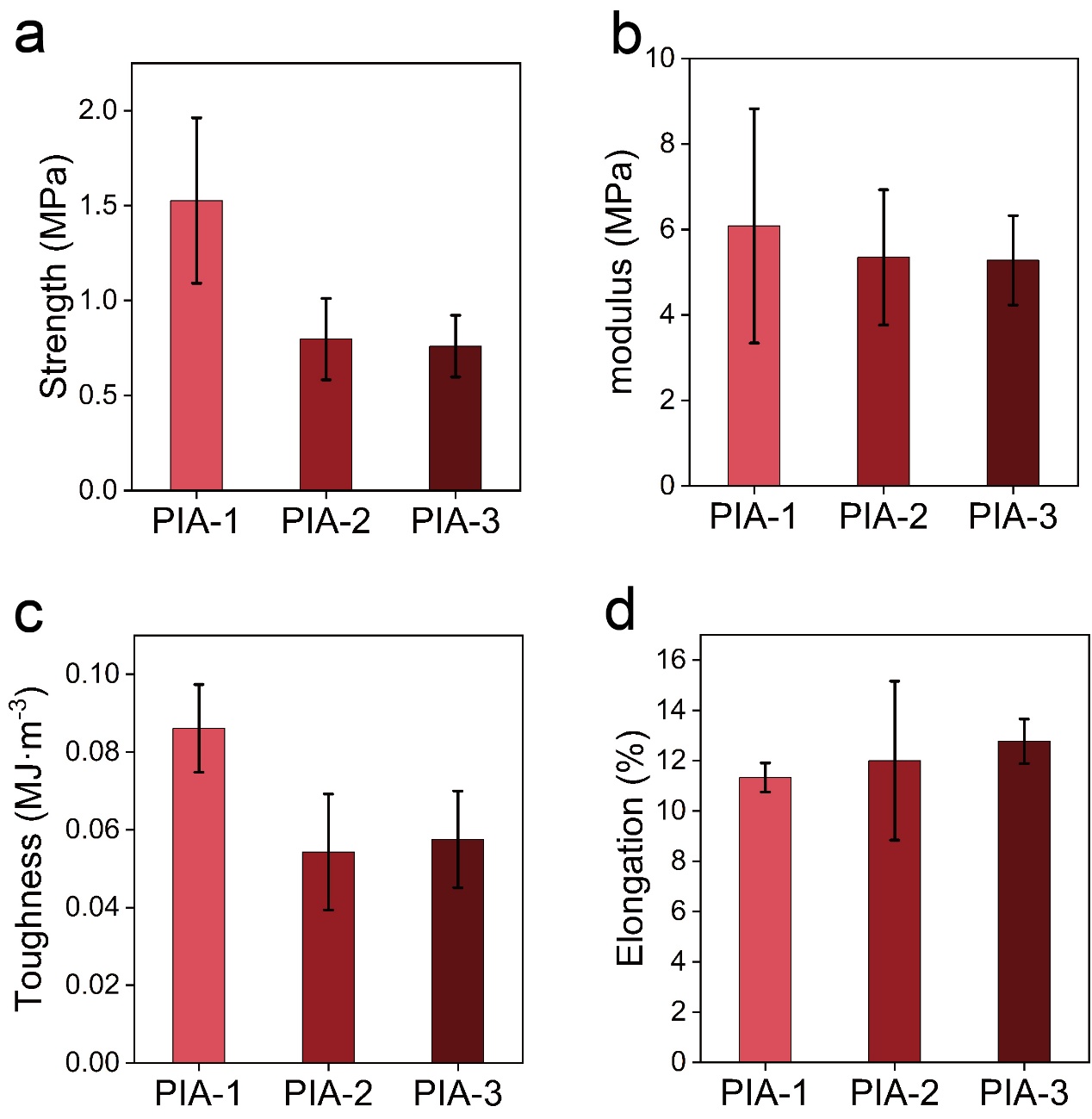


**Figure S3.** (a) Tensile strength, (b) Young’s modulus, (c) Toughness and (d) Elongation comparison of PIA-1, PIA-2 and PIA-3.

**
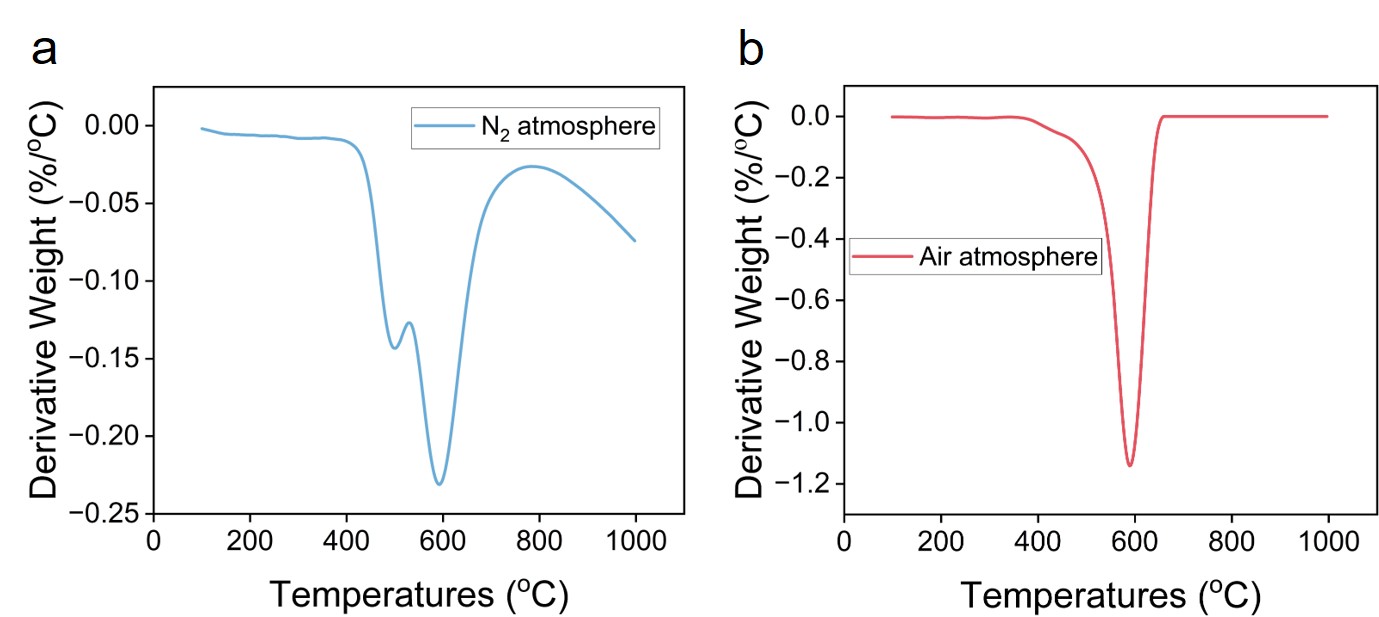
**

**Figure S4.** DTG curves of PIA under (a) nitrogen and (b) air atmospheres.


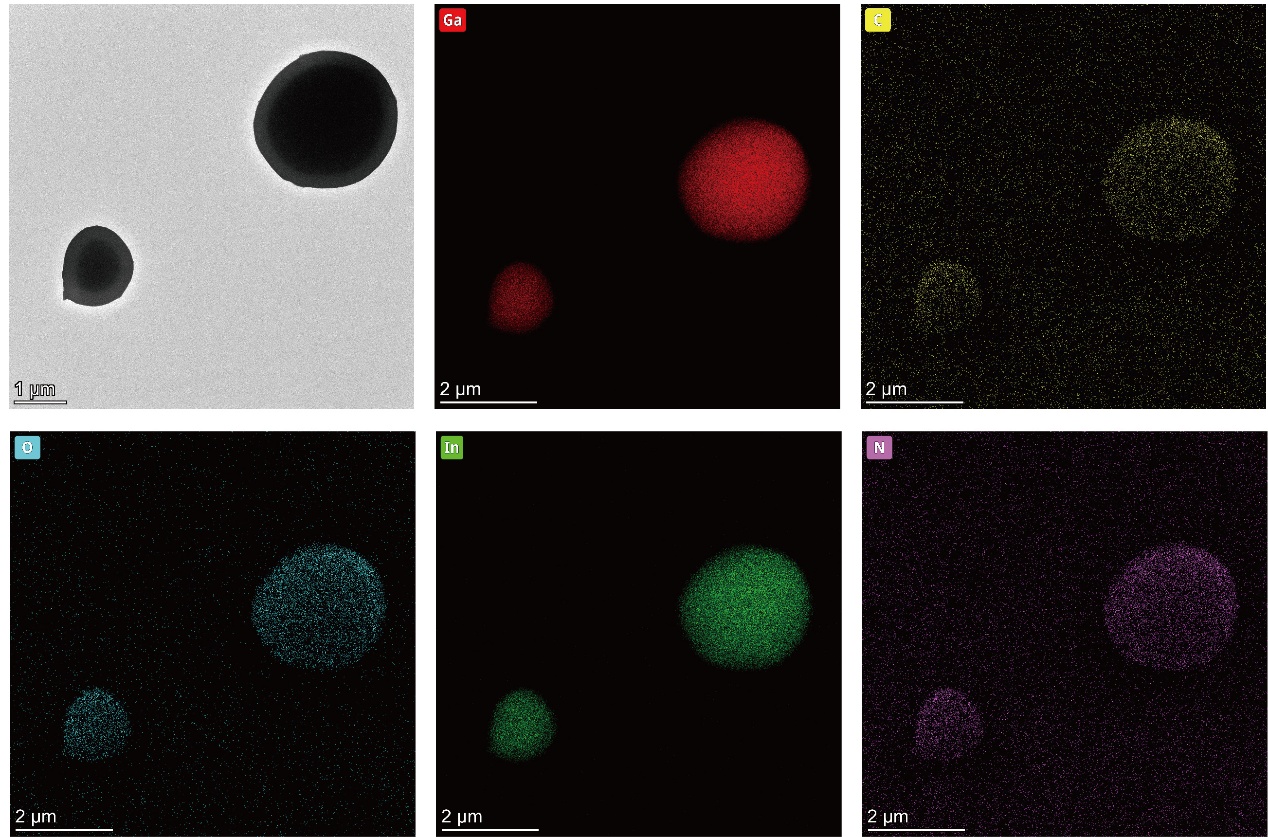


**Figure S5.** Elemental mapping images of PAA encapsulated LM particles.


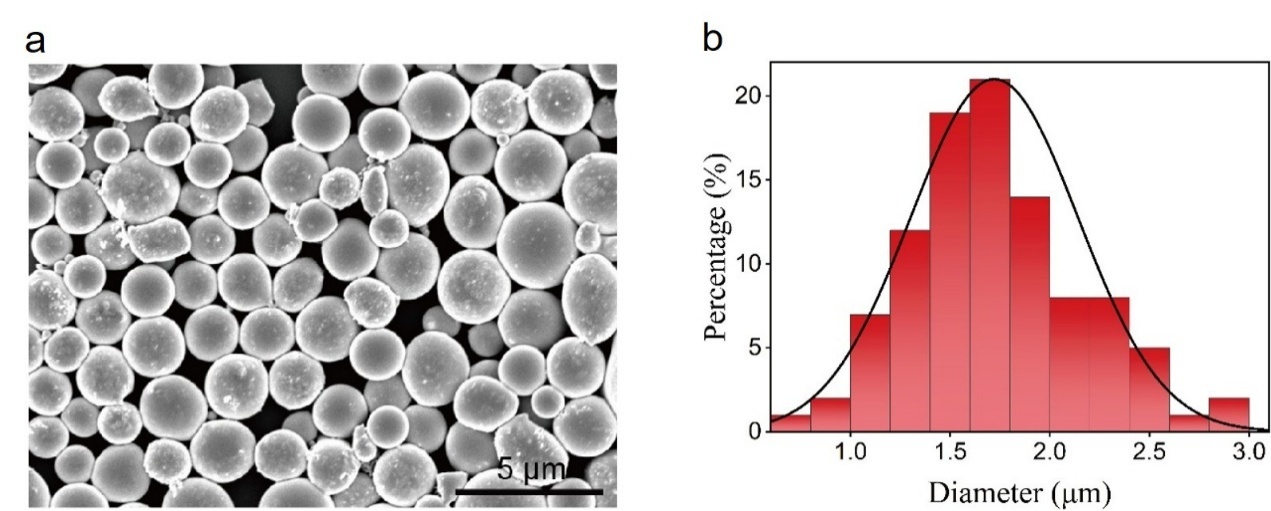


**Figure S6.** SEM image of (a) PAA coated LM particles and corresponding (b) particle size distribution.


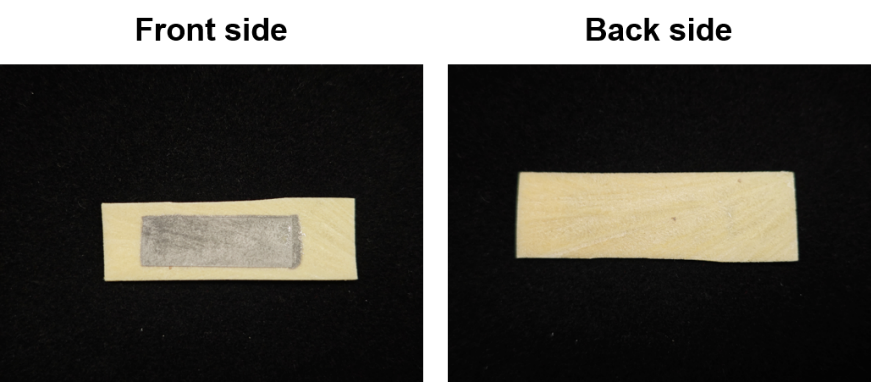


**Figure S7.** Digital images of the front and back sides of the PIA substrate after printing liquid metal ink.


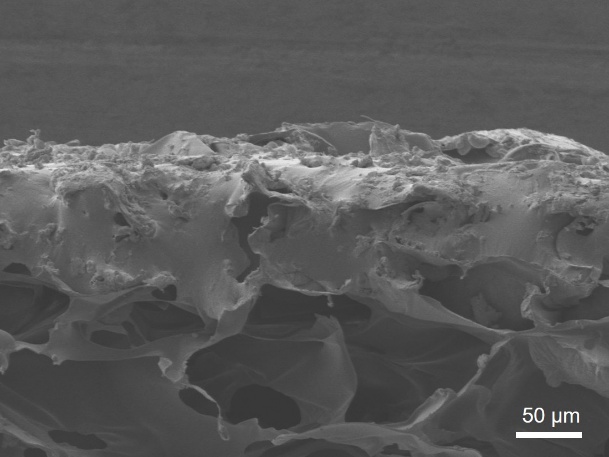


**Figure S8.** The SEM image of the cross-sectional morphology of CIPA.


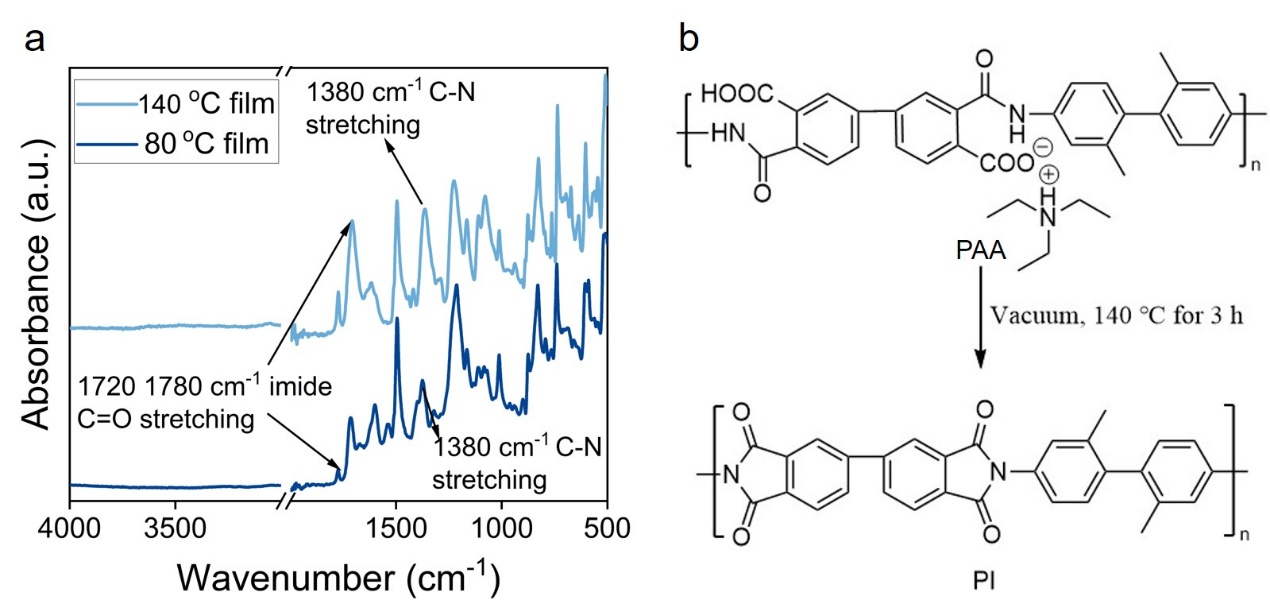


**Figure S9.** (a) FTIR spectra of PAA films after imidization at 80 °C and 140 °C. (b) Thermal imidization process from poly (amic acid) to polyimide.


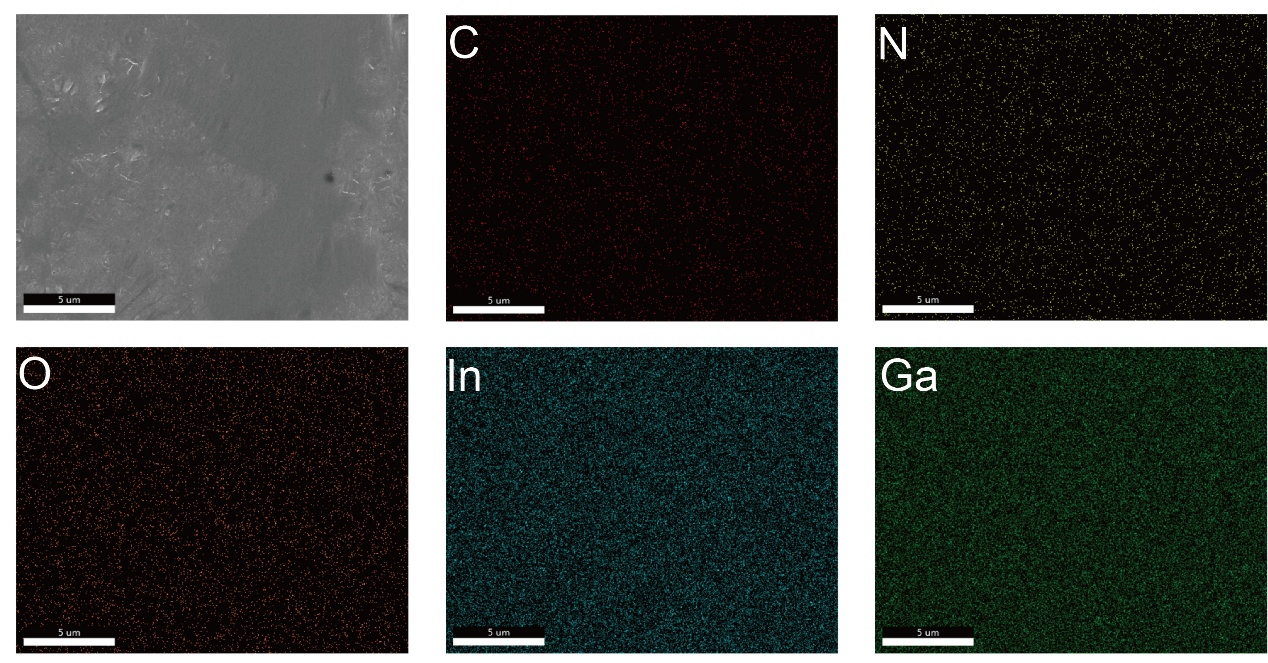


**Figure S10.** SEM energy-dispersive spectroscopy (EDS) spectra of the PAA coated liquid metal ink after thermal sintering.


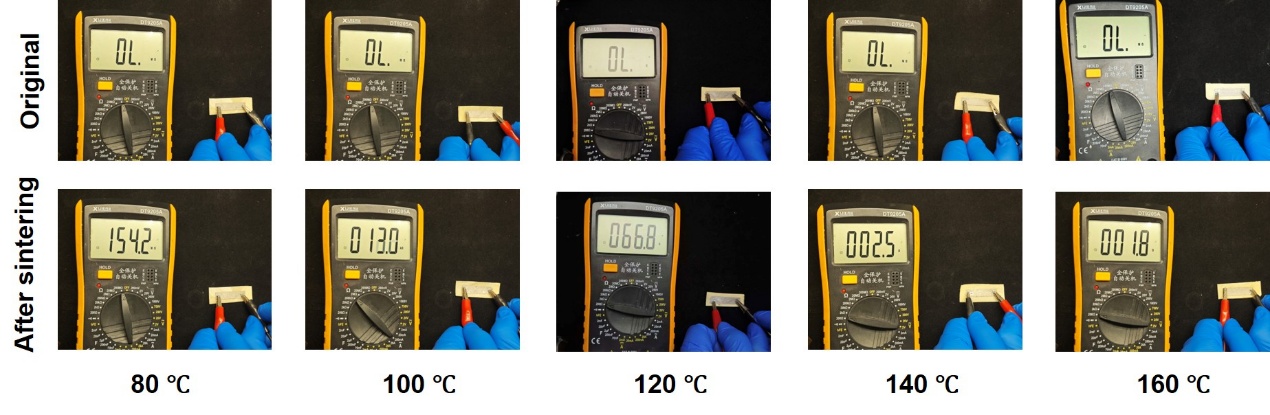


**Figure S11.** Digital images of surface two-wire resistance of liquid metal ink printed on polyimide aerogels before and after thermal sintering at different sintering temperatures.


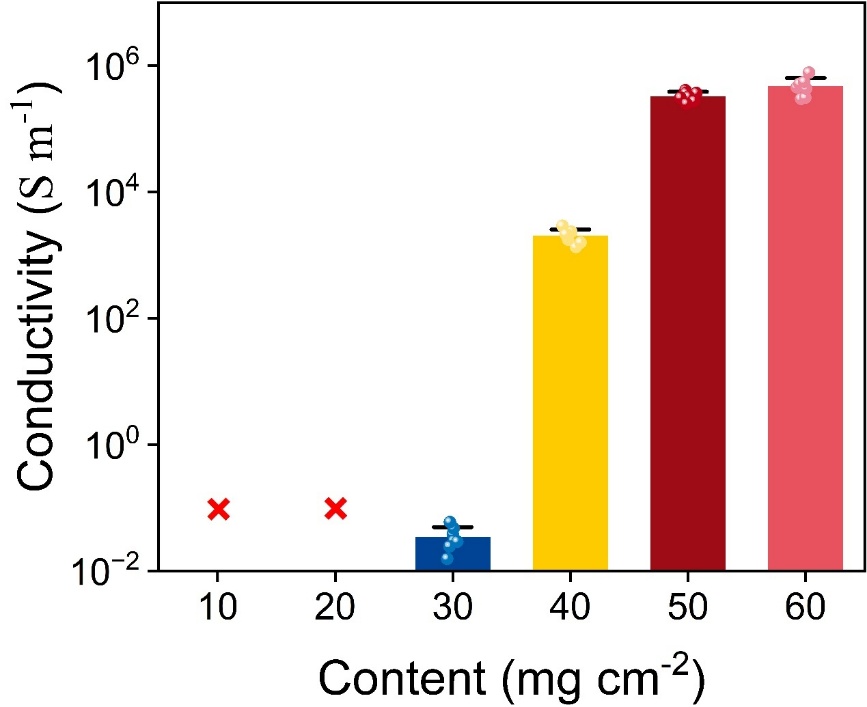


**Figure S12.** Influence of liquid metal ink dosage on electrical conductivity.

**
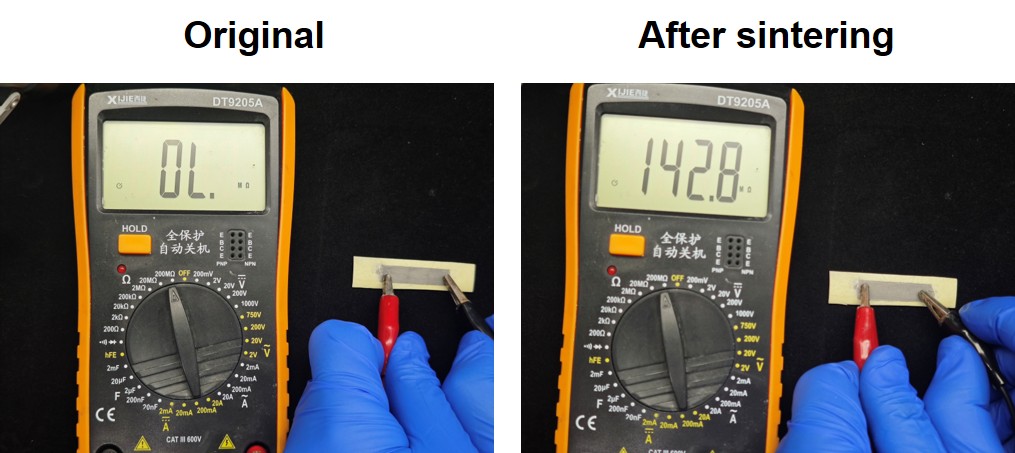
**

**Figure S13.** Resistance comparison of pure liquid metal particles after thermal sintering


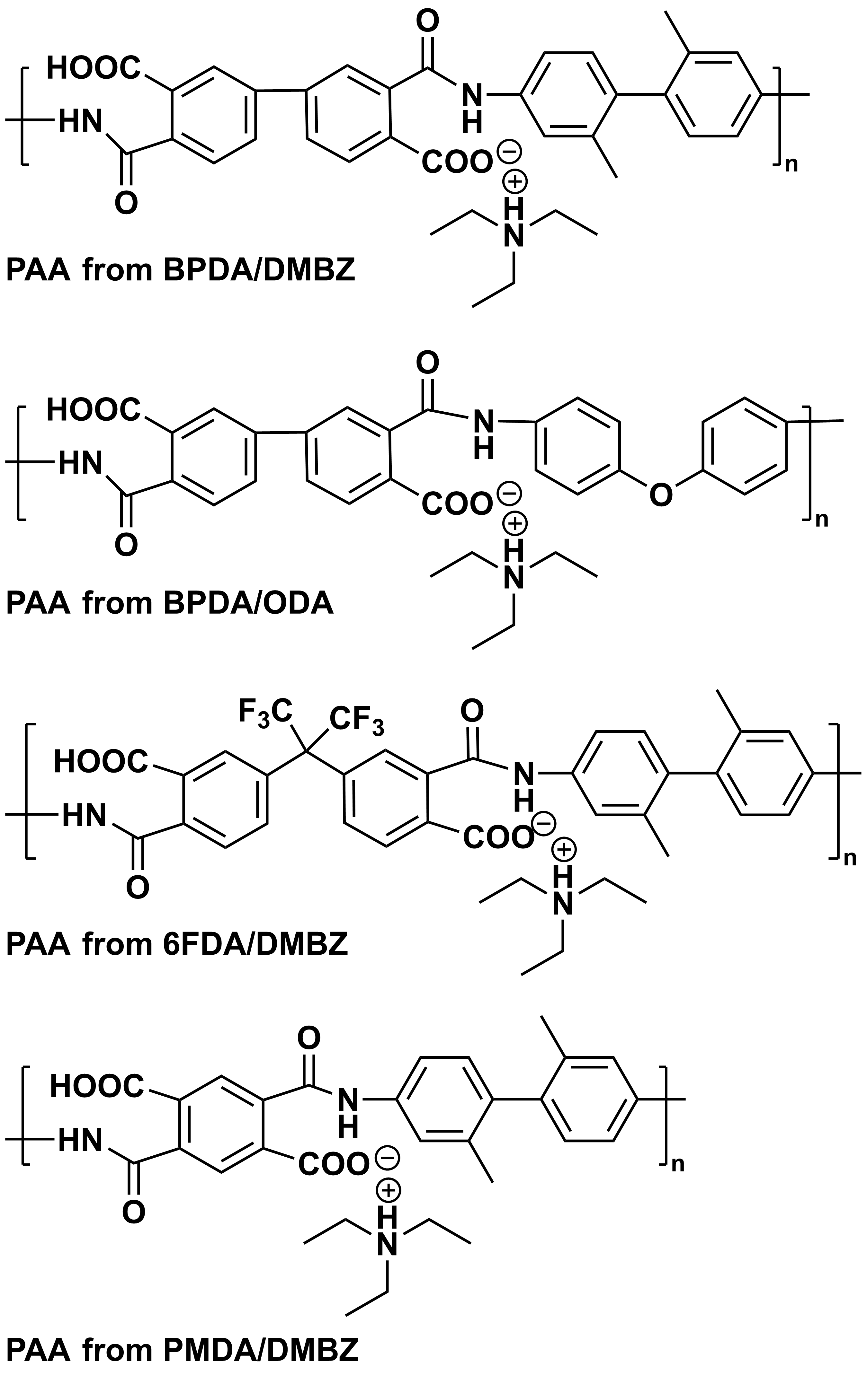


**Figure S14.** Chemical structures of PAA derived from BPDA/DMBZ, BPDA/ODA, 6FDA/DMBZ, and PMDA/DMBZ.


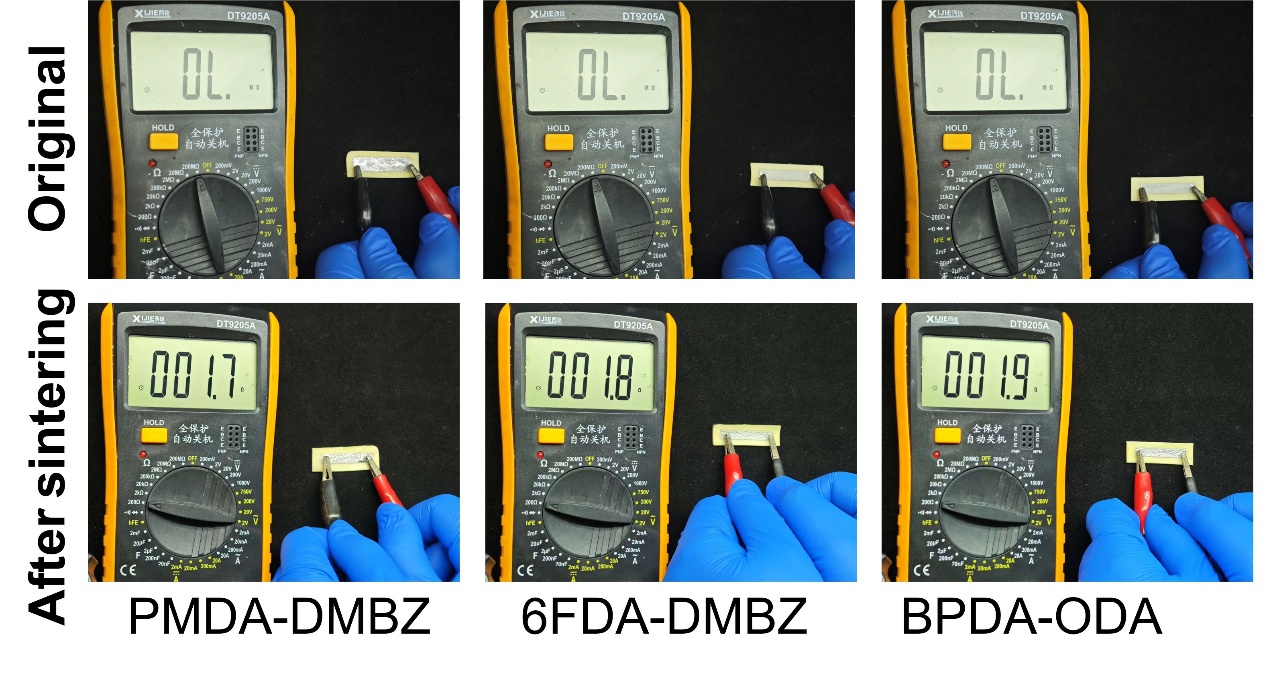


**Figure S15.** Digital images of surface resistance of liquid metal inks prepared from PMDA/DMBZ, 6FDA/DMBZ and BPDA/ODA monomers, printed on PIA substrates before and after thermal sintering.


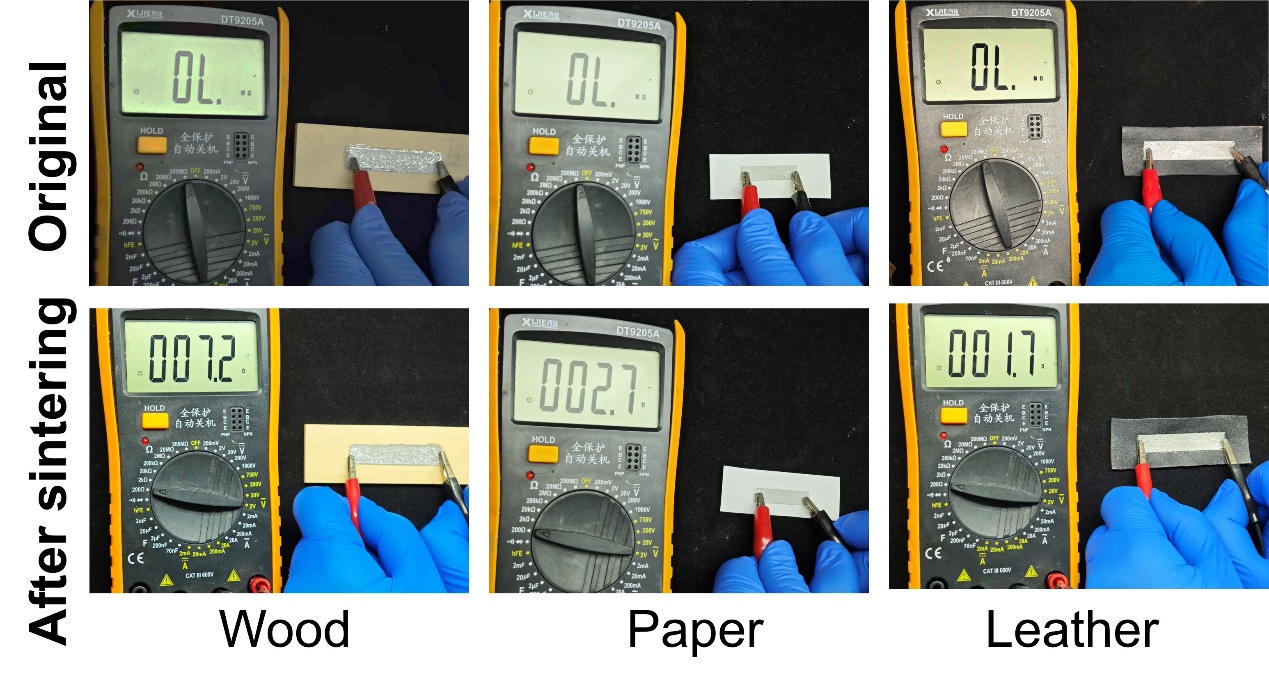


**Figure S16.** Digital images of surface resistance of PAA engineered liquid metal ink printed on wood, paper, and leather after thermal imidization.


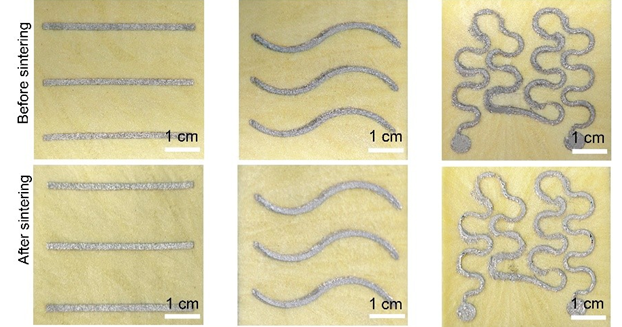


**Figure S17.** Digital images of PAAP patterns printed on PIA substrates, the upper images show the state before thermal sintering, and the lower images show the activated state.


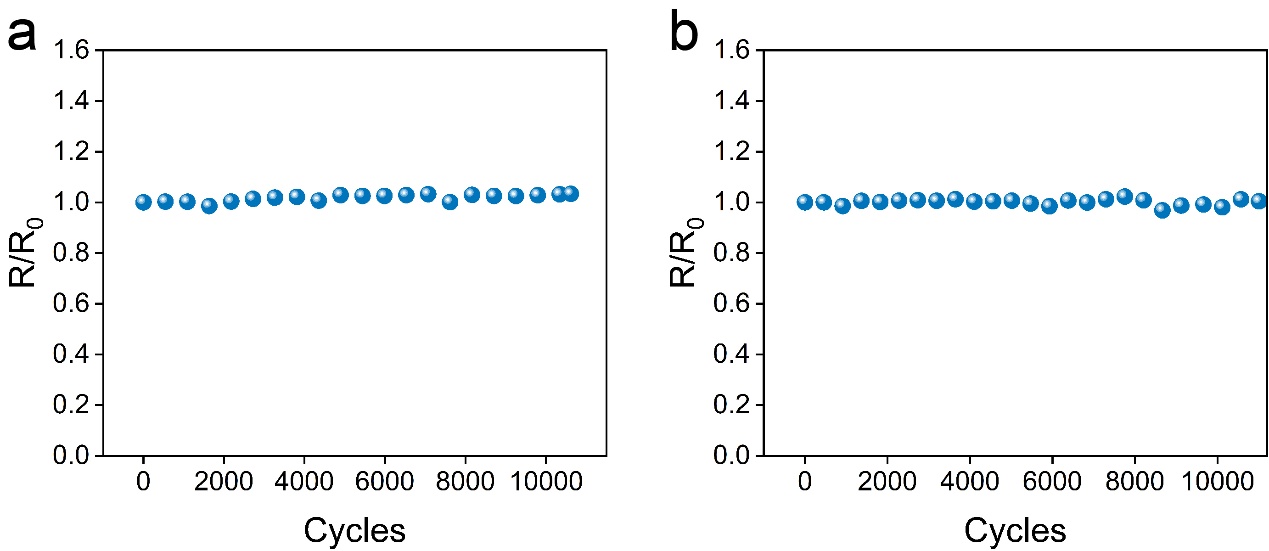


**Figure S18.** a) Resistance change rate of PAAP printed on leather under bending cycles (bending radius: 1–10 mm, cycle duration: 10000). b) Resistance change rate of PAAP printed on paper (bending radius: 1–10 mm, cycle duration: 10000).


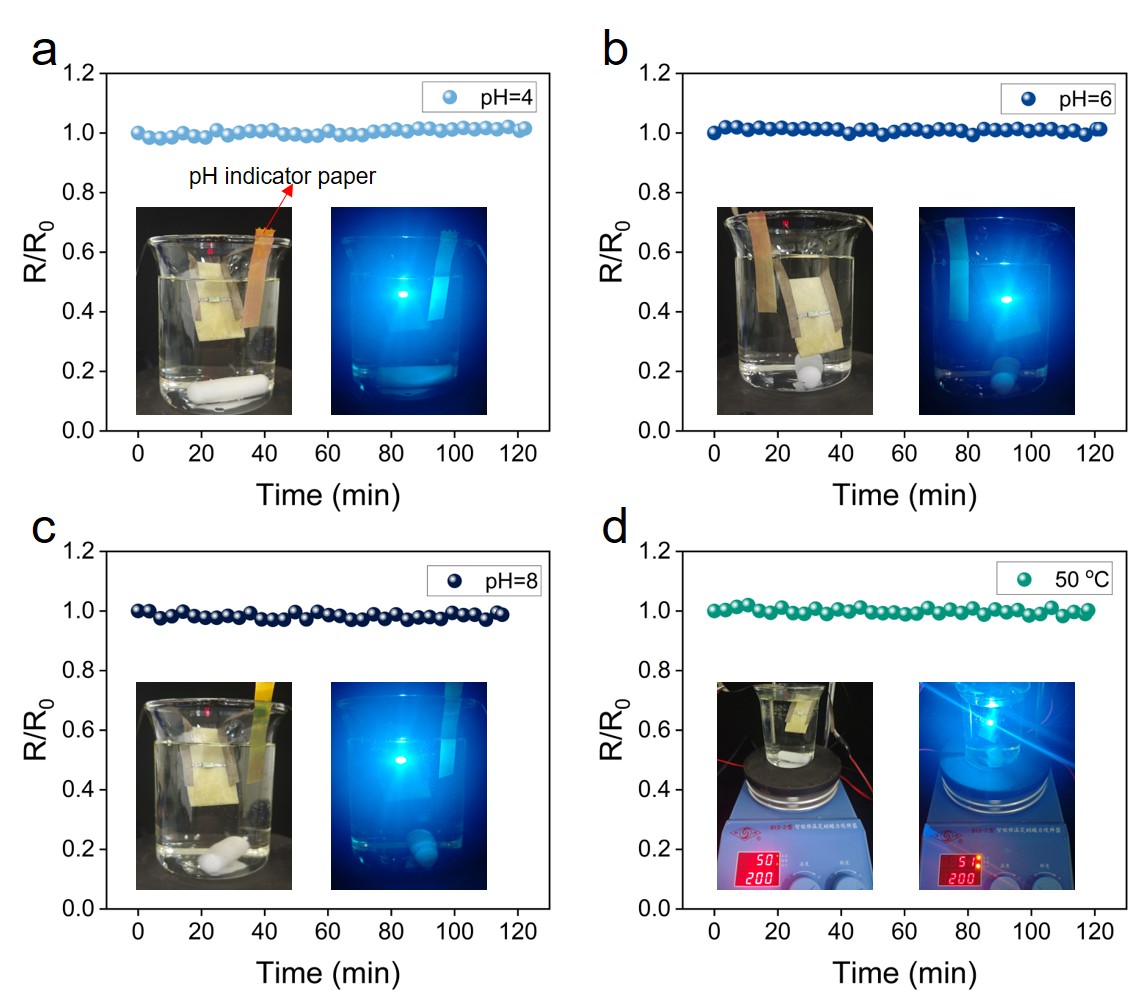


**Figure S19.** (a-c) Resistance stability of CIPA after 120 min immersion under continuous stirring in artificial sweat solutions (pH 4, 6, and 8). (d) Resistance stability of CIPA after 120 min immersion under continuous stirring in water at 50 °C.

**
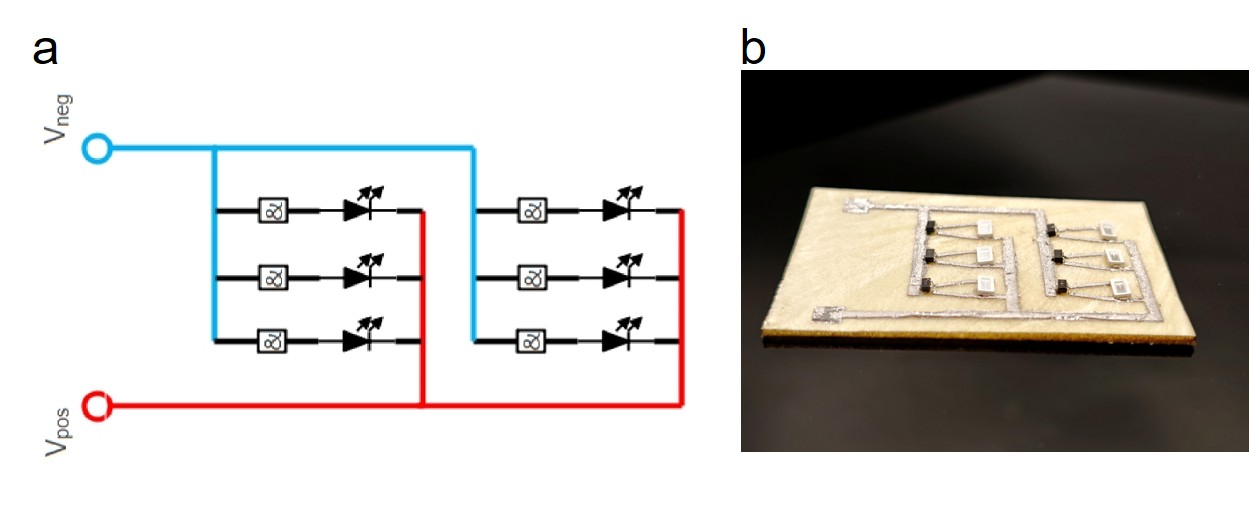
**

**Figure S20.** (a) Diagram of Hall sensor circuit. (b) Digital photo of Hall sensor based on PIA.


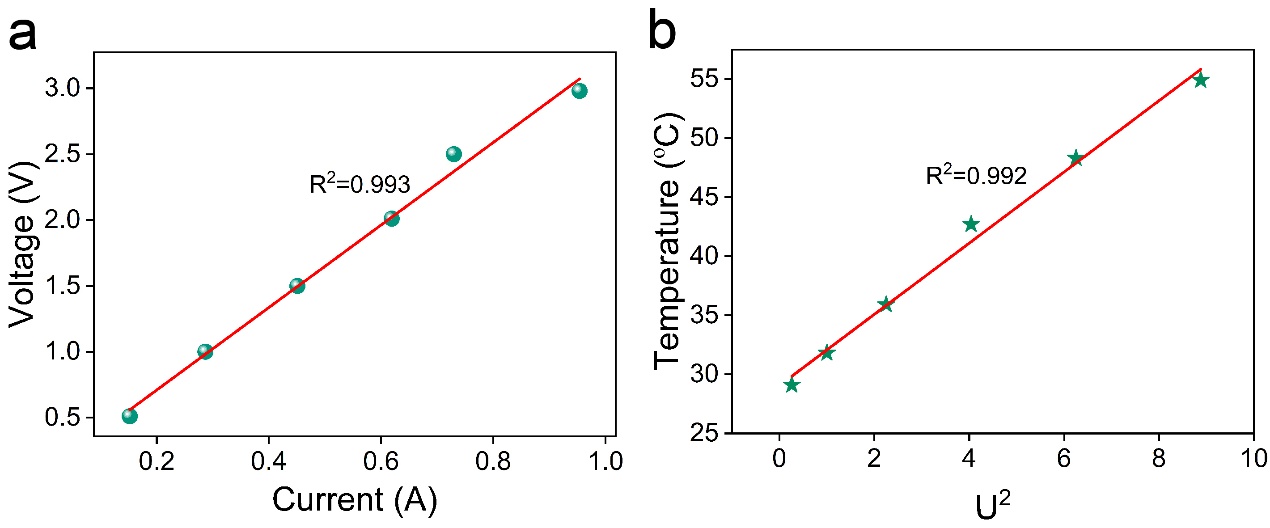


**Figure S21.** (a) Voltage-current curve and (b) of Temperature-U² curve of aerogel Joule heaters.


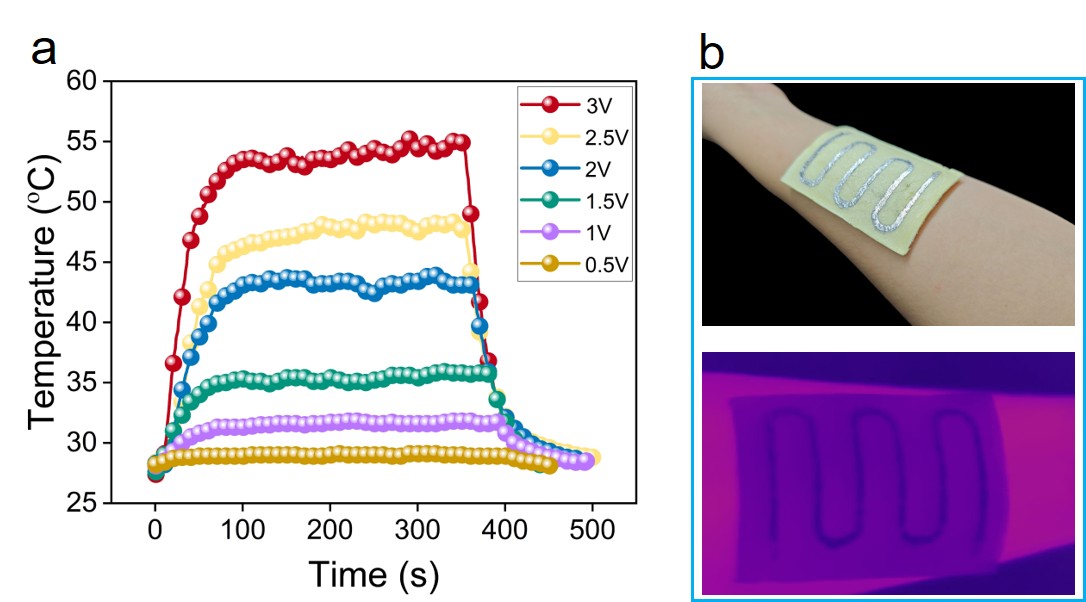


**Figure S22.** (a) Stepwise temperature rise curves at 0–3 V. (b) Practical application demonstration of the aerogel Joule heater on the arm.


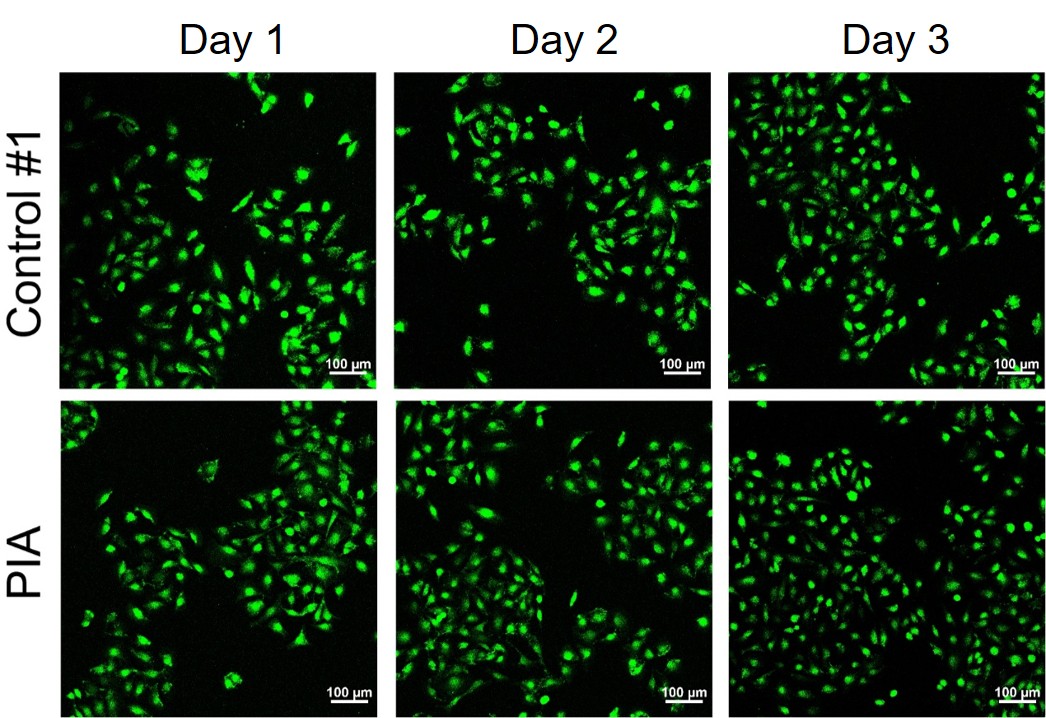


**Figure S23.** Bright-field images and fluorescence images of cells cultured on PIA substrate in medium.


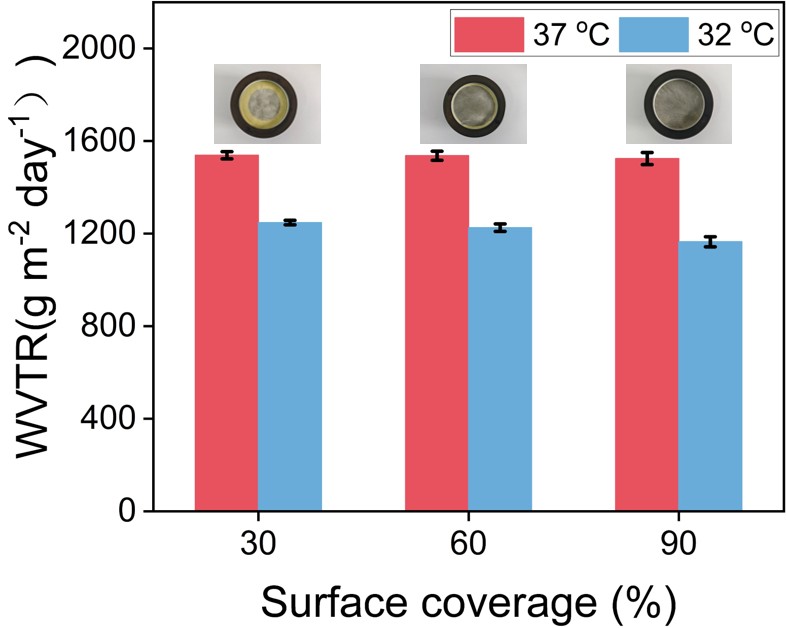


**Figure S24.** Comparison of WVTR of CIPA with different (PAAP) coverage ratios (30%, 60%, and 90%) at 32 °C and 37 °C.


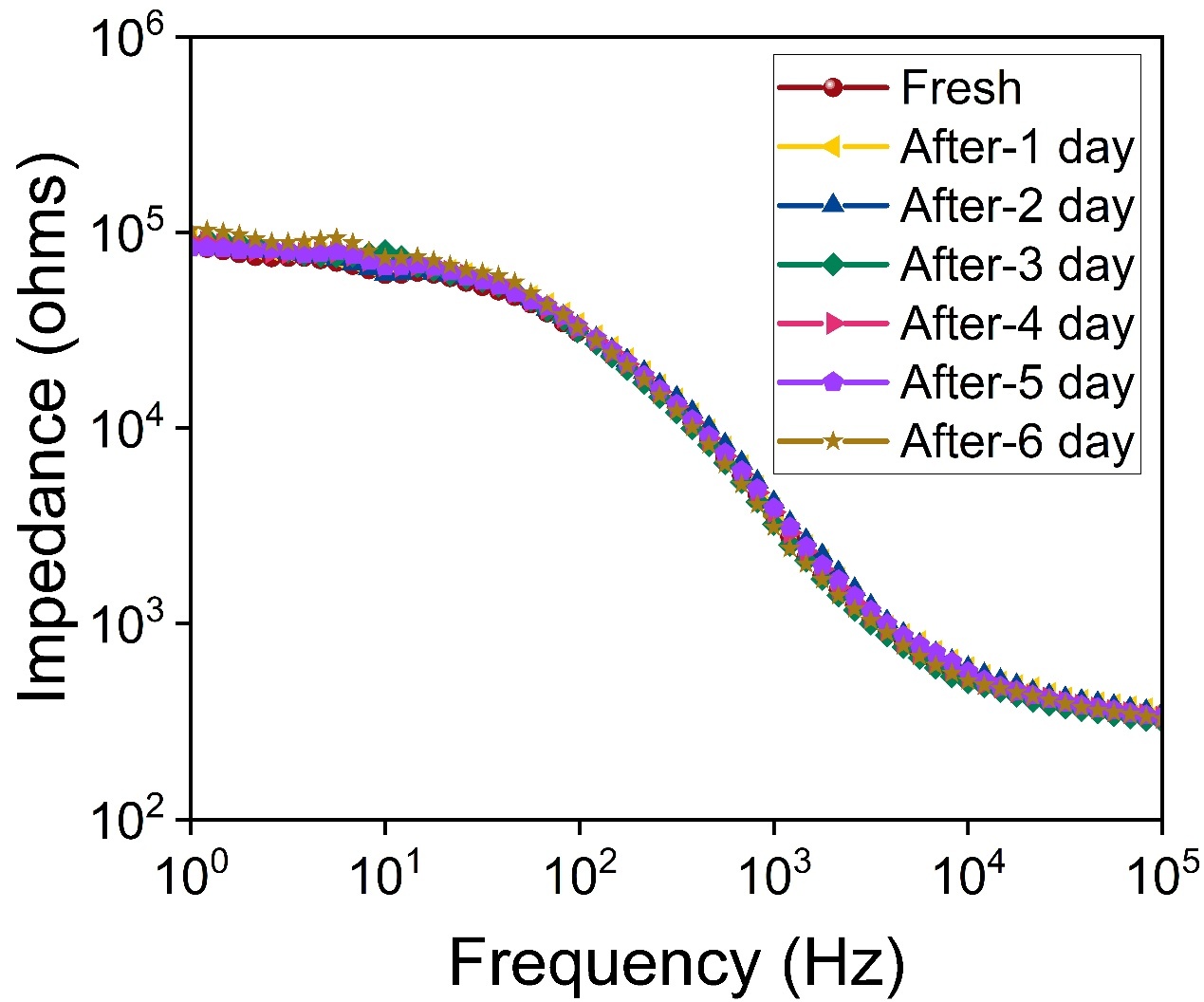


**Figure S25.** Interfacial impedance of the CIPA electrodes during a 7-day wearing.


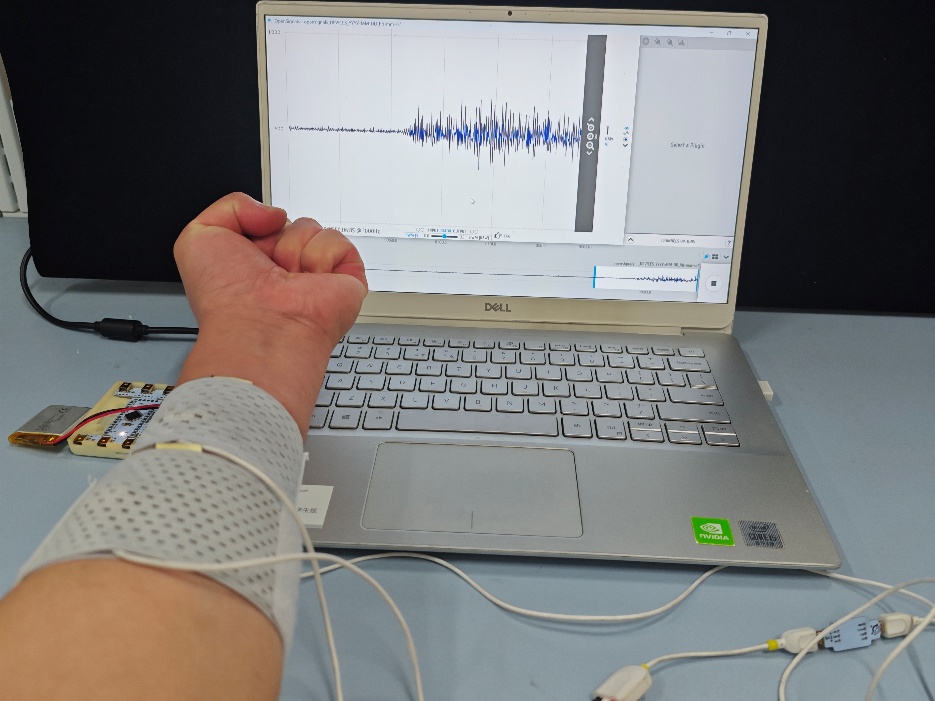


**Figure S26.** Digital images of CIPA electrodes applied for EMG recording.

**
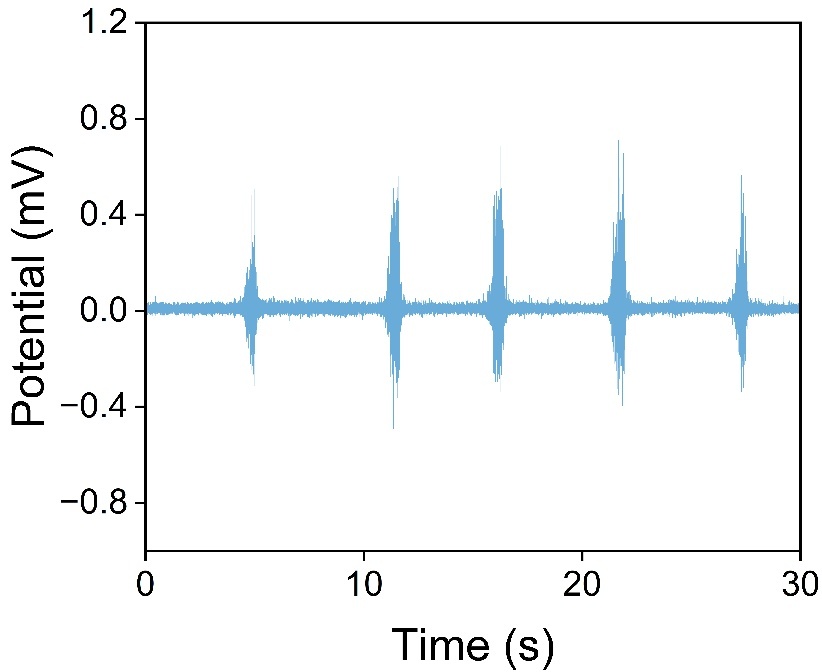
**

**Figure S27.** Underwater EMG signal recordings.


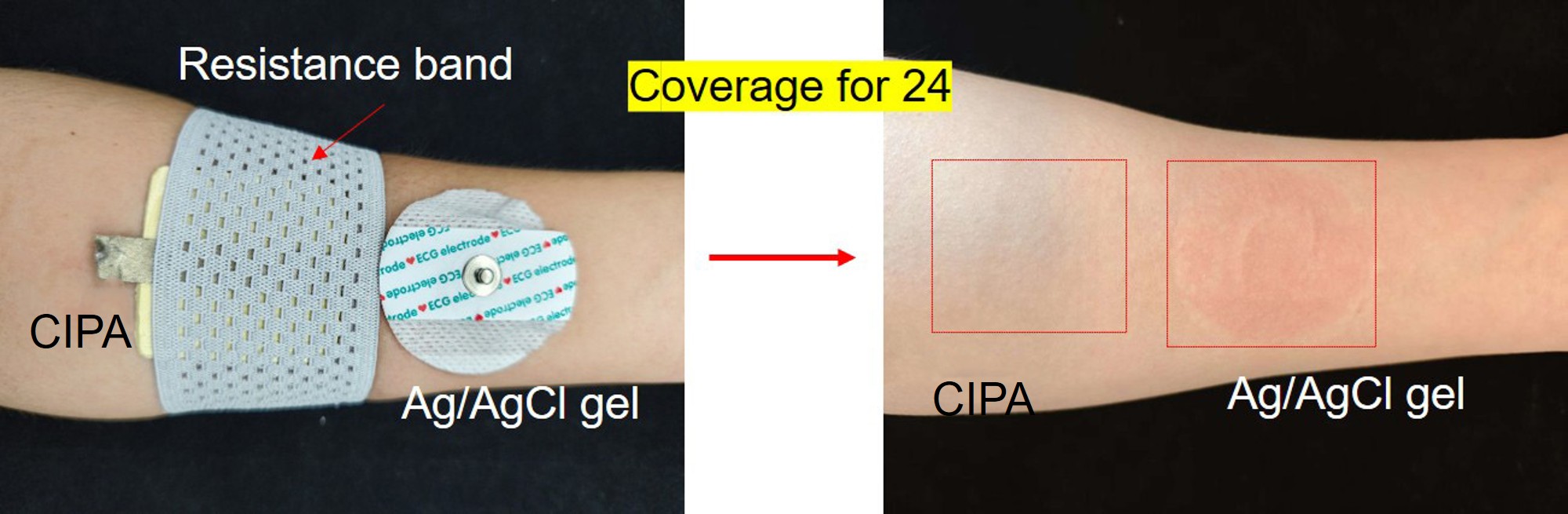


**Figure S28.** Digital photos of human skin with commercial Ag/AgCl gel and CIPA worn for 24 hours.


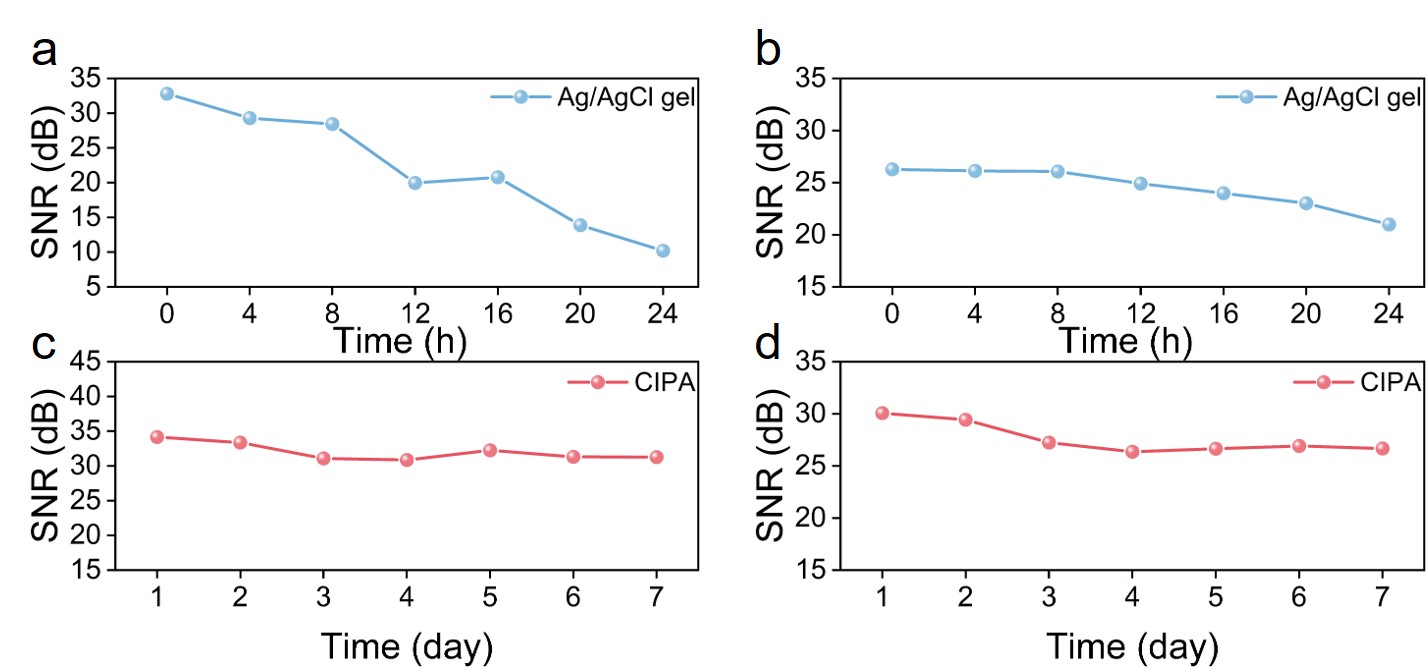


**Figure S29.** SNR values: (a) EMG signal and (b) ECG amplitude during a 24-hour wearing using Ag/AgCl electrodes. (c) EMG signal and (d) ECG amplitude during a 7-day wearing using commercial CIPA E-skin.


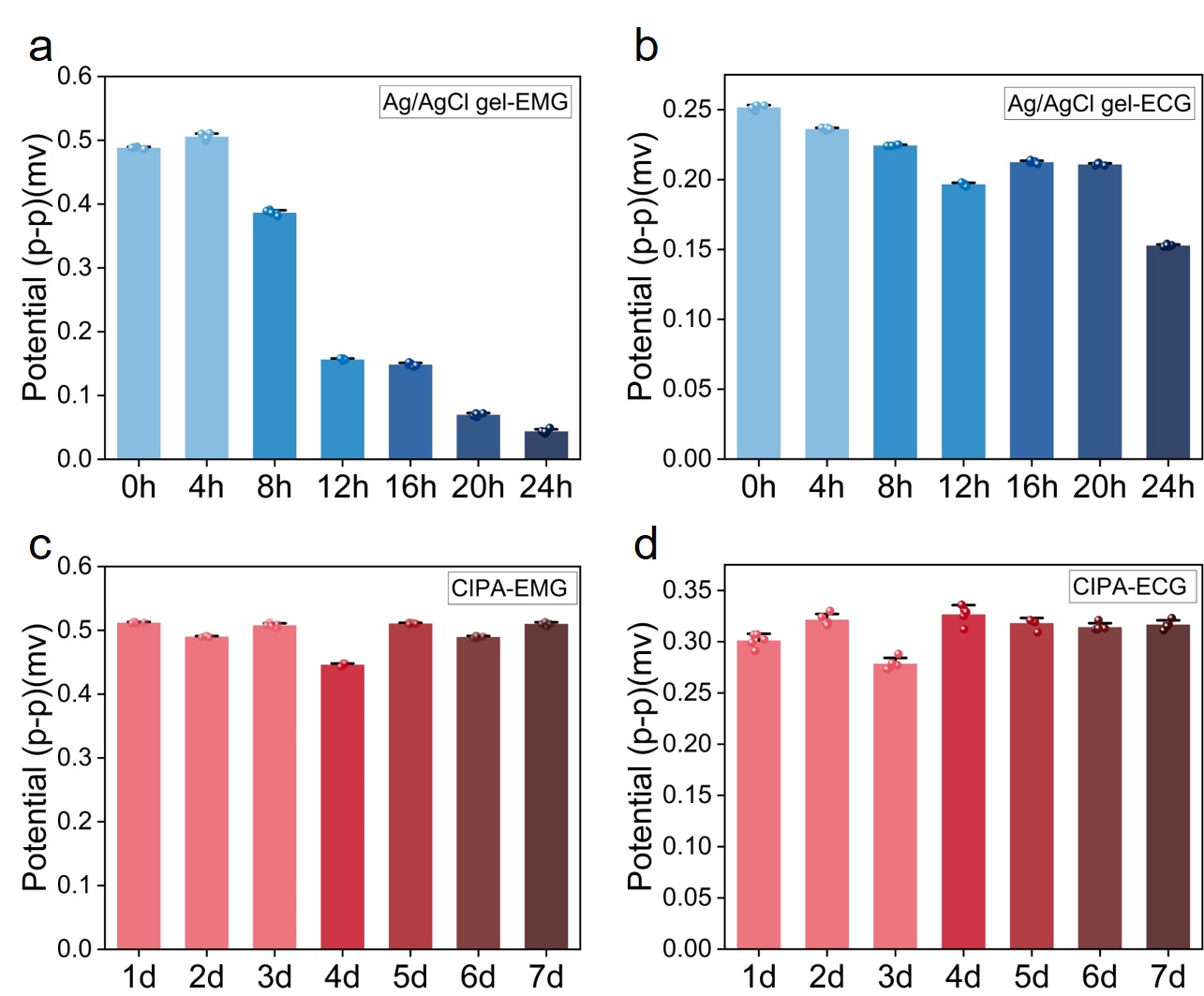


**Figure S30.** (a) EMG signal and (b) ECG amplitude during a 24-hour wearing using commercial Ag/AgCl electrodes. (c) EMG signal and (d) ECG amplitude during a 7-day wearing using CIPA electrodes.

**Supplementary Tables**

**Table S1.** Thermal stability analysis.

| Atmosphere | T_d5%_(℃) | T_d10%_（℃） | Charcoal residue (%) |
| --- | --- | --- | --- |
| Air | 462 | 512 | 7 |
| N_2_ | 477 | 506 | 49 |

**Table S2.** Comparison of advantages of PAAP with different liquid metal ink sintering methods

| Sintering method | Universality | Conductivity  (S m^-1^) | Sintering efficiency | Application scope | Line stability | Ref |
| --- | --- | --- | --- | --- | --- | --- |
| Mechanical Sintering | low | 1.64×10^5^ | low | high | low | ^[3]^ |
| Mechanical Sintering | high | 9×10^5^ | low | high | low | ^[4]^ |
| Thermal Sintering | low | 1.25×10^4^ | low | low | high | ^[5]^ |
| Laser Sintering | high | 8.65×10^5^ | low | high | high | ^[6]^ |
| Laser Sintering | high | 3.625×10^5^ | low | low | high | ^[7]^ |
| Electrochemical Sintering | high | 5.0×10^5^ | low | low | high | ^[8]^ |
| Chemical Sintering | high | 1.2×10^5^ | low | low | high | ^[9]^ |
| Evaporation Induced Sintering | low | 2×10^5^ | high | low | low | ^[10]^ |
| Field-Based Alignment Sintering | low | 2.55×10^5^ | low | low | low | ^[11]^ |
| Freezing-Activated Sintering | low | 5.6×10^4^ | low | low | low | ^[12]^ |
| **Thermal Sintering** | **high** | **8.2×10^5^** | **high** | **high** | **high** | **This work** |

**Table S3.** Comparison of strain stability of different conductive inks

| Materials | MAX stain (%) | R/R_0_ | Ref. |
| --- | --- | --- | --- |
| S80-conductor | 70 | 6 | ^[13]^ |
| LM/PU | 500 | 3 | ^[14]^ |
| LM/PDMS | 100 | 3.725 | ^[15]^ |
| DN ion gel | 500 | 12.5 | ^[16]^ |
| GNP-w-CNT | 150 | 19 | ^[17]^ |
| Ag NW ink | 7.1 | 30 | ^[18]^ |
| Ag NPs | 100 | 50 | ^[19]^ |
| PDMSOH-DTB | 100 | 3.75 | ^[15]^ |
| CNT/PU | 300 | 4.2 | ^[20]^ |
| Au | 115 | 20.2 | ^[21]^ |
| CNT/PDMS | 150 | 4 | ^[22]^ |
| **PAAP** | **600** | **2.177** | **This work** |

**Table S4.** Comparison of water vapor transmission rate (WVTR) for different porous polymer substrates.

| Materials | WVTR (g m^-2^ day^-1^) | Ref. |
| --- | --- | --- |
| SET | 150 | ^[23]^ |
| MXene @ SA/PLA | 527.3 | ^[24]^ |
| SGFM | 528 | ^[25]^ |
| In-textile | 561 | ^[26]^ |
| FIBHT | 657 | ^[27]^ |
| Janus TPU | 663 | ^[28]^ |
| P3D-eskin | 676 | ^[29]^ |
| BreaCARES | 700 | ^[30]^ |
| LeCA | 790 | ^[31]^ |
| WADE-skin | 852 | ^[32]^ |
| **PIA** | **1700** | **This work** |

**Legends for movies S1 to S5**

**Movie S1.** The process of pure liquid metal sliding down on polyimide aerogel

**Movie S2.** Polyimide aerogel permeability demonstration

**Movie S3.** Brightness changes of the LED lights of the Hall sensor under different magnetic field intensities

**Movie S4.** The output voltage of the Hall sensor changes as the distance between it and the magnet gets closer

**Movie S5.** The process of using CIPA to monitor human electromyographic (EMG) signals

**References**

[1] S. Plimpton, *J. Comput. Phys.* **1995**, *117*, 1.

[2] L. Sanchez-Botero, D. S. Shah, R. Kramer-Bottiglio, *Adv Mater* **2022**, *34*, e2109427.

[3] V. Vallem, V. Aggarwal, M. D. Dickey, *Advanced Materials Technologies* **2022**, *8*.

[4] L. Yu, X. Qi, Y. Liu, L. Chen, X. Li, Y. Xia, *ACS Appl Mater Interfaces* **2022**, *14*, 48150.

[5] S. Liu, S. N. Reed, M. J. Higgins, M. S. Titus, R. Kramer-Bottiglio, *Nanoscale* **2019**, *11*, 17615.

[6] C. Cho, W. Shin, M. Kim, J. Bang, P. Won, S. Hong, S. H. Ko, *Small* **2022**, *18*, e2202841.

[7] S. Liu, M. C. Yuen, E. L. White, J. W. Boley, B. Deng, G. J. Cheng, R. Kramer-Bottiglio, *ACS Appl Mater Interfaces* **2018**, *10*, 28232.

[8] Z. Lin, X. Qiu, Z. Cai, J. Li, Y. Zhao, X. Lin, J. Zhang, X. Hu, H. Bai, *Nat Commun* **2024**, *15*, 4806.

[9] Z. Song, J. Ma, R. Fang, J. Liu, H. Wang, *ACS Applied Electronic Materials* **2024**, *6*, 3884.

[10] S. Lee, S. A. Jaseem, N. Atar, M. Wang, J. Y. Kim, M. Zare, S. Kim, M. D. Bartlett, J. W. Jeong, M. D. Dickey, *Chem Rev* **2025**, *125*, 3551.

[11] T. T. Hoang, P. T. Phan, M. T. Thai, J. Davies, C. C. Nguyen, H.-P. Phan, N. H. Lovell, T. N. Do, *Advanced Intelligent Systems* **2022**, *4*.

[12] H. Wang, Y. Yao, Z. He, W. Rao, L. Hu, S. Chen, J. Lin, J. Gao, P. Zhang, X. Sun, X. Wang, Y. Cui, Q. Wang, S. Dong, G. Chen, J. Liu, *Adv. Mater.* **2019**, *31*, e1901337.

[13] H. S. Lee, Y. Jo, J. H. Joo, K. Woo, Z. Zhong, S. Jung, S. Y. Lee, Y. Choi, S. Jeong, *ACS Appl Mater Interfaces* **2019**, *11*, 12622.

[14] R.-m. Zheng, Y.-h. Wu, Y.-h. Xu, S.-q. Liu, H.-z. Liu, P.-p. Wang, Z.-f. Deng, S. Chen, L. Liu, *Materials Letters* **2019**, *235*, 133.

[15] Y. Wang, J. Li, L. Sun, H. Chen, F. Ye, Y. Zhao, L. Shang, *Adv Mater* **2023**, *35*, e2211731.

[16] J. Lan, B. Zhou, C. Yin, L. Weng, W. Ni, L.-Y. Shi, *Polymer* **2021**, *231*.

[17] F. Zhang, D. Ren, L. Huang, Y. Zhang, Y. Sun, D. Liu, Q. Zhang, W. Feng, Q. Zheng, *Advanced Functional Materials* **2021**, *31*.

[18] J. Liang, K. Tong, Q. Pei, *Adv Mater* **2016**, *28*, 5986.

[19] N. Matsuhisa, D. Inoue, P. Zalar, H. Jin, Y. Matsuba, A. Itoh, T. Yokota, D. Hashizume, T. Someya, *Nat Mater* **2017**, *16*, 834.

[20] M. K. Shin, J. Oh, M. Lima, M. E. Kozlov, S. J. Kim, R. H. Baughman, *Adv Mater* **2010**, *22*, 2663.

[21] Y. Kim, J. Zhu, B. Yeom, M. Di Prima, X. Su, J. G. Kim, S. J. Yoo, C. Uher, N. A. Kotov, *Nature* **2013**, *500*, 59.

[22] D. J. Lipomi, M. Vosgueritchian, B. C. Tee, S. L. Hellstrom, J. A. Lee, C. H. Fox, Z. Bao, *Nat Nanotechnol* **2011**, *6*, 788.

[23] J. Gao, M. Hu, H. Sun, Y. Wang, Y. Wei, W. Li, L. Zheng, M. Xu, Q. Lu, Z. Liu, H. Yang, Y. Wang, W. Song, X. Wang, W. Huang, *npj Flexible Electronics* **2025**, *9*.

[24] S. Ding, X. Jin, J. Guo, B. Kou, M. Chai, S. Dou, G. Jin, H. Zhang, X. Zhao, J. Ma, X. Li, X. Liu, B. Wang, X. Zhang, *Advanced Fiber Materials* **2024**, *7*, 156.

[25] J. Jiang, X. Song, Y. Qi, X. Tao, Z. Zheng, Q. Huang, *Advanced Fiber Materials* **2025**, *7*, 894.

[26] P. Wang, X. Ma, Z. Lin, F. Chen, Z. Chen, H. Hu, H. Xu, X. Zhang, Y. Shi, Q. Huang, Y. Lin, Z. Zheng, *Nat Commun* **2024**, *15*, 887.

[27] K. Yao, Q. Zhuang, Q. Zhang, J. Zhou, C. K. Yiu, J. Zhang, D. Ye, Y. Yang, K. W. Wong, L. Chow, T. Huang, Y. Qiu, S. Jia, Z. Li, G. Zhao, H. Zhang, J. Zhu, X. Huang, J. Li, Y. Gao, H. Wang, J. Li, Y. Huang, D. Li, B. Zhang, J. Wang, Z. Chen, G. Guo, Z. Zheng, X. Yu, *Science Advances* **2024**, *10*, eadq9575.

[28] Y. Ni, B. Li, C. Chu, S. Wang, Y. Jia, S. Cao, R. E. Neisiany, C. He, S. Chen, Z. You, *Sci Bull (Beijing)* **2025**, *70*, 712.

[29] Q. Zhuang, K. Yao, C. Zhang, X. Song, J. Zhou, Y. Zhang, Q. Huang, Y. Zhou, X. Yu, Z. Zheng, *Nature Electronics* **2024**, *7*, 598.

[30] Q. Zhuang, K. Yao, X. Song, Q. Zhang, C. Zhang, H. Wang, R. Yang, G. Zhao, S. Li, H. Shu, Q. Huang, Y. Chai, X. Yu, Z. Zheng, *Science Advances* **2025**, *11*, eadu3146.

[31] M. Qiang, X. Wang, B. Cheng, W. He, X. Huang, G. Huang, L. Wang, S. K. Ravi, X. Yao, *Advanced Functional Materials* **2025**.

[32] F. Chen, Q. Zhuang, Y. Ding, C. Zhang, X. Song, Z. Chen, Y. Zhang, Q. Mei, X. Zhao, Q. Huang, Z. Zheng, *Adv Mater* **2023**, *35*, e2305630.
